# Current Management of Pheochromocytoma/Paraganglioma: A Guide for the Practicing Clinician in the Era of Precision Medicine

**DOI:** 10.3390/cancers11101505

**Published:** 2019-10-08

**Authors:** Svenja Nölting, Martin Ullrich, Jens Pietzsch, Christian G. Ziegler, Graeme Eisenhofer, Ashley Grossman, Karel Pacak

**Affiliations:** 1 Department of Medicine IV, University Hospital, LMU Munich, Ziemssenstraße 1, 80336 München, Germany; 2 Department of Radiopharmaceutical and Chemical Biology, Institute of Radiopharmaceutical Cancer Research, Helmholtz-Zentrum Dresden-Rossendorf, Bautzner Landstrasse 400, 01328 Dresden, Germany; m.ullrich@hzdr.de (M.U.); j.pietzsch@hzdr.de (J.P.); 3 Department of Chemistry and Food Chemistry, School of Science, Technische Universität Dresden, Mommsenstrasse 9, 01062 Dresden, Germany; 4 Department of Medicine III, University Hospital Carl Gustav Carus Dresden, Fetscherstraße 74, 01307 Dresden, Germany; Christian.Ziegler@uniklinikum-dresden.de (C.G.Z.); Graeme.Eisenhofer@uniklinikum-dresden.de (G.E.); 5 Institute for Clinical Chemistry and Laboratory Medicine, University Hospital Carl Gustav Carus at Technische Universität Dresden, 01307 Dresden, Germany; 6 Oxford Centre for Diabetes, Endocrinology and Metabolism, University of Oxford, Oxford Ox3 7LJ, UK; ashley.grossman@ocdem.ox.ac.uk; 7 Department of Gastroenterology, Royal Free Hospital ENETS Centre of Excellence, London NW3 2QG, UK; 8 *Eunice Kennedy Shriver* National Institute of Child Health and Human Development, National Institutes of Health, Bethesda, MD 20814, USA; karel@mail.nih.gov

**Keywords:** pheochromocytoma, paraganglioma, guideline, genetics, diagnosis, biomarkers, imaging, follow-up, therapy, precision medicine

## Abstract

Pheochromocytomas and paragangliomas (PCC/PGLs) are rare, mostly catecholamine-producing neuroendocrine tumors of the adrenal gland (PCCs) or the extra-adrenal paraganglia (PGL). They can be separated into three different molecular clusters depending on their underlying gene mutations in any of the at least 20 known susceptibility genes: The pseudohypoxia-associated cluster 1, the kinase signaling-associated cluster 2, and the Wnt signaling-associated cluster 3. In addition to tumor size, location (adrenal vs. extra-adrenal), multiplicity, age of first diagnosis, and presence of metastatic disease (including tumor burden), other decisive factors for best clinical management of PCC/PGL include the underlying germline mutation. The above factors can impact the choice of different biomarkers and imaging modalities for PCC/PGL diagnosis, as well as screening for other neoplasms, staging, follow-up, and therapy options. This review provides a guide for practicing clinicians summarizing current management of PCC/PGL according to tumor size, location, age of first diagnosis, presence of metastases, and especially underlying mutations in the era of precision medicine.

## 1. Introduction

Pheochromocytomas/paragangliomas (PCCs/PGLs) are rare neuroendocrine tumors, mostly catecholamine-producing and originating from chromaffin tissue derived from the neural crest. The incidence of diagnosed PCC/PGL is about 0.8/100,000 patients/year [1] with around 30–40% being hereditary and another 40–50% of patients showing identifiable somatic mutations in many of the currently identified 20 PCC/PGL susceptibility genes [2,3]. However, the incidence may be underestimated, since earlier studies showed that 50% of PCCs/PGLs found at autopsy were not clinically suspected or diagnosed [4]. 

The mean age of PCC/PGL diagnosis is the fourth and fifth decade, although 10–20% of all cases are diagnosed in children [5]. Patients with known mutations in susceptibility genes tend to develop PCC/PGL at a younger age, compared with patients with sporadic tumors, in part because of biochemical and/or anatomic surveillance.

PCCs arise from the adrenal medulla; PGLs are extra-adrenal and originate from the sympathetic paraganglia (85% arise below the diaphragm) or from the parasympathetic paraganglia (head-and-neck paragangliomas). All PGLs arising from the parasympathetic ganglia are denoted as head-and-neck (HN) PGLs, although they may also arise from the anterior and middle mediastinum along the vagus nerve. Over 95% of HN PGLs are non-functioning and do not overproduce noradrenalin, although a small number (1–3%) of HN PGLs can overproduce it. PCCs and PGLs may produce adrenalin (epinephrine), noradrenalin (norepinephrine), or dopamine in varying proportions, depending on adrenal versus extra-adrenal location and the underlying gene mutation as well as developmental background (sympathetic vs. parasympathetic). 

Measurement of free metanephrine, normetanephrine, and 3-methoxytyramine—the respective O-methylated metabolites of epinephrine, norepinephrine, and dopamine—provides currently the best recommended plasma test whereas measurement of the two first metabolites, preferably in the free form but also after acid hydrolysis as deconjugated metabolites, provides recommended urinary tests for PCC/PGL diagnosis [6] with recent evidence of the slight superiority of the plasma over urinary tests [6]. However, 3-methoxytyramine may not be available in the United States and other countries in commercial laboratories, although its assessment is recommended, where possible. Tumoral secretion of catecholamines leads to signs and symptoms of migraine-like headache, sweating, palpitations, episodic or sustained hypertension, anxiety, tremor, nausea, weakness, pallor, weight loss, or postural hypotension. These features are, however, non-specific, and vary according to tumoral catecholamine production, content, and secretion; this can be independent of tumor size, explaining why some similarly-sized tumors produce symptoms while others do not [7]. Yet, in intra-patient comparisons, increase of tumor diameter and tumor burden correlates with the increase in plasma and urinary metanephrines but less accurately with urinary catecholamines [8]. Very rarely, PCCs/PGLs may be non-functional and synthesize no or only negligible catecholamines reaching a large size before diagnosis, usually due to their mass effect [9]; however, most cases of PCCs/PGLs with negative test results for plasma O-methylated metabolites reflect small tumors or metastatic disease with some degree of cell dedifferentiation [6]. 

About 10% of PCCs and a significantly higher proportion—35–40%—of PGLs are metastatic, especially those due to mutations of the succinate dehydrogenase A/B (*SDHA/B*) gene [10,11,12,13,14,15,16,17]. The median five-year survival rate of metastatic patients is around 60–70% but this differs according to the populations studied [18]. Malignancy is defined by the presence of distant metastases at sites where chromaffin cells are normally absent—bones and lymph nodes [19]. However, for lung and liver, it has to be considered that both organs normally contain ganglia, and therefore in these locations, it may be difficult to differentiate between metastases and primary PGLs, especially if past medical history is negative [20,21,22,23,24,25]. Validated prognostic pathological parameters for malignant PCCs/PGLs are lacking, although some risk stratification systems have been described, such as *The Pheochromocytoma of the Adrenal Gland Scaled Score* (PASS) [26] and the more recently extended *Grading of Adrenal Pheochromocytoma and Paraganglioma* (GAPP) [27]. However, as reviewed [28], these score systems have limited predictive ability. Nevertheless, the ’rule-out’ value of both algorithms seems promising in order to exclude future metastatic potential: PCCs with a PASS score <4 and PCCs/PGLs with a GAPP score <3 are exceedingly seldom malignant [29].

In the most recent American Joint Committee of Cancer Staging (AJCC) guidelines a novel TNM staging for PCC/PGL was introduced, and clinical relevance has recently been validated retrospectively with PCCs and sympathetic PGLs in stage III–IV (regional lymph node metastases or invasion into surrounding tissue or distant metastases) being associated with increased PASS/GAPP scores and increased mortality, as well as aberrations in the pseudo-hypoxia pathway cluster [30,31].

Moreover, there are some predictors associated with a higher likelihood of metastatic disease. These include size (≥5–6 cm), extra-adrenal location of the primary tumor, noradrenergic/dopaminergic biochemical phenotype, mutations of the succinate dehydrogenase A and B (*SDHA/B*) genes, tumor multiplicity/recurrence, and age at first presentation (<20 years) [5,17]. This review summarizes the more recent findings concerning biomarkers and their application in a clinical setting, up-to-date imaging modalities, follow-up recommendations for apparently sporadic and hereditary PCC/PGLs, and therapeutic approaches including surgery and treatments for malignancy. 

## 2. Genetics

Around 30–40% of PCC/PGL patients show identifiable germline and another 46% somatic mutations in at least 20 known susceptibility genes [2,32,33]. This means that around three-quarters of all PCC/PGL patients can be assigned to one of the three molecular clusters (as recently reviewed [33,34]).

### 2.1. Cluster 1: Pseudohypoxic Krebs Cycle-Related Genes and Pseudohypoxia VHL/EPAS1-Related Genes 

**Cluster 1 pseudohypoxic Krebs cycle-related genes**: genes encoding succinate dehydrogenase subunits (*SDHx [SDHA, SDHB, SDHC, SDHD]*), succinate dehydrogenase complex assembly factor-2 (*SDHAF2*), fumarate hydratase (*FH*), malate dehydrogenase 2 (*MDH2*), and isocitrate dehydrogenase 1 (*IDH1*); **cluster 1 pseudohypoxia *VHL/EPAS1*-related genes:** Egl-9 prolyl hydroxylase-1 and -2 (*PHD1/2*), von Hippel–Lindau tumor suppressor (*VHL*) and hypoxia-inducible factor 2α (*HIF2A/EPAS1/2*).

Cluster 1 is called pseudohypoxic since it mimics cellular hypoxia: The Krebs cycle is disrupted due to mutations in several genes (*SDHA[AF2]/B/C/D, FH, MDH2*, and *IDH*) and, therefore, the Krebs cycle and oxidative phosphorylation are impaired resulting in increased cellular glycolysis. This in turn leads to accumulation of the oncometabolites succinate, fumarate, or 2-hydroxyglutarate which favors DNA hypermethylation and inactivation of different tumor suppressor genes, including *PHD1/2*, as previously reviewed [35]. Inactivation of *PHD1/2* results in less HIF-α hydroxylation and less HIF-α ubiquitination/degradation (HIF-α is stabilized), which is also VHL-dependent; thus, *VHL* mutations also lead to HIF-α stabilization and accumulation. Via HIF-α stabilization and accumulation, cluster 1 mutations promote angiogenesis (VEGF/PDGF transcription amongst others), tumor invasion, metastasis and other cellular processes. HIF-α is the confluent of cluster 1 mutations, and interconnects cluster 1 with cluster 2 mutations [36]. PCCs/PGLs resulting especially from mutations in Krebs cycle enzyme *SDHx* subunits are often multiple, aggressive, metastatic, and have a poorer prognosis, compared to PCCs/PGLs bearing other susceptibility gene mutations, especially those related to cluster 2 [37]. Cluster 1 mutations can be subdivided in pseudohypoxic Krebs cycle-related and pseudohypoxia *VHL/EPAS1*-related PCCs/PGLs (see above). Almost all tumors belonging to this cluster have a noradrenergic biochemical phenotype and produce noradrenalin (measured metabolite: normetanephrine), and/or dopamine (measured metabolite: 3-methoxytyramine), but no adrenalin (measured metabolite: metanephrine).

### 2.2. Cluster 2: Kinase Signaling-Related Genes

Genes belonging to cluster 2 are the rearranged-during-transfection (*RET*) proto-oncogene, neurofibromin 1 (*NF1*) tumor suppressor, *HRAS*, transmembrane protein 127 (*TMEM127*), and Myc-associated factor X (*MAX*). These mutations lead to activation of the phosphatidylinositol-3-kinase (PI3K)/AKT, mammalian target of rapamycin (mTORC1)/p70S6 kinase (p70S6K), and RAS/RAF/ERK signaling pathway and promote cell proliferation, survival, cancer development, and angiogenesis. Most of the cluster 2 mutations lead to an adrenergic biochemical phenotype with production of adrenalin (measured metabolite: metanephrine) +/− noradrenalin (measured metabolite: normetanephrine). *Max* mutations are most likely to show an intermediate biochemical phenotype [38].

### 2.3. Cluster 3: Wnt Signaling-Related Genes

The Cold Shock Domain-containing E1 gene (*CSDE1*) and the ’mastermind-like’ transcriptional coactivator 3 (*MAML3*) fusion genes belong to cluster 3 [2]. *MAML3* mutations lead to overactivation of Wnt and Hedgehog signaling. These tumors strongly express chromogranin A. *CSDE1* mutations lead to over-activation of β-catenin, a target of Wnt signaling, and favor tumor proliferation, invasion, and metastases. The catecholamine phenotype is not known. *MAML3*-mutated PCCs/PGLs showed high Ki-67 expression, aggressive behavior, and early metastatic spread [2].

### 2.4. Summary: Cluster 1, 2 and 3

Knowledge about how a particular patient may be assigned to one of these clusters can guide biochemical testing, specific imaging modalities, and appropriate personalized treatments (Table 1) [32]. 

This further justifies subjecting all PCC/PGL patients to genetic testing for germline mutations [39].

Somatic tumor mutations may indeed also influence prognosis, since different somatic mutations correlate with different metastatic risks in a similar manner as germline mutations do (Table 1), although, of course, this is still the subject of ongoing research. Moreover, a personalized molecular-targeted therapy approach could result from somatic mutations such as *RET, NF1, HRAS, MAX, TMEM127*, or those of defective pseudohypoxia signaling, much as it does from the molecular testing in other cancer entities such as differentiated thyroid carcinoma, bronchial carcinoma, or breast cancer [40,41].

#### Conclusion/Practical Tips 

We recommend genetic testing for germline mutations for *all* PCC/PGL patients; testing of tumor material, where available, for somatic tumor mutation analysis can also provide useful additional information. 

## 3. Diagnosis

### 3.1. Biochemistry 

Measurements of plasma free or urinary metanephrines are the recommended screening tests for the biochemical diagnosis of PCC/PGL [42], with recent evidence now establishing the superiority of the plasma over urinary tests [6]. However, high accuracy of the plasma test can only be assured using appropriately established measurement methods and reference intervals, combined with correctly applied preanalytics, especially sampling of blood in the supine position [6,43,44,45]. Under these conditions, plasma concentrations of normetanephrine, metanephrine, or methoxytyramine more than two-fold above upper cut-offs of reference intervals indicate a high probability of PCC/PGL even at low pre-test prevalence of disease [8,46]. Combined increases of two or more metabolites also suggest a high probability of PCC/PGL. In other cases, intra-patient longitudinal comparisons can be useful to confirm progression of disease (i.e. increased disease burden), which in most cases is slow and involves a doubling-time of over two years [8]. Biochemical testing for PCC/PGL in patients screened due to signs and symptoms of apparent catecholamine excess should always be performed before imaging [42]. Moreover, it is important to instruct the patients to abstain from caffeine, black tea, nicotine, alcohol, bananas, cheese, almonds, nuts, chocolate, eggs, or vanilla three days prior to assessment of plasma free or urine metanephrines. Tricyclic antidepressants, serotonin re-uptake inhibitors, ephedrine, cocaine, and metamphetamine may lead to false positive results.

As shown in Table 1, pseudohypoxic cluster 1 type PCC/PGL are linked to a noradrenergic (predominantly increased normetanephrine) phenotype because the tumors lack the enzyme, phenylethanolamine N-methyl transferase (PNMT), which converts noradrenalin to adrenalin [47,48]. Moreover, a substantial proportion of PCCs/PGLs in *SDHx* mutation carriers also appear to show some additional deficiency in the enzyme dopamine-β-hydroxylase, which catalyzes the final step in catecholamine synthesis—the conversion of dopamine to noradrenalin; patients with such tumors show increased production of the dopamine metabolite 3-methoxytyramine [8,49,50]. Thus, all cluster 1 PCCs/PGLs may be diagnosed by elevated normetanephrine levels, with or without increases in 3-methoxytyramine. However, measurements of urinary 3-methoxytyramine are *not* useful and only plasma 3-methoxytyramine is of significant diagnostic utility [6]. Moreover, *SDHx* mutation carriers often show reduced synthesis and secretion of normetanephrine and in rare cases lack the initial rate-limiting enzyme of catecholamine synthesis (tyrosine hydroxylase); such cases may be termed non-functional or biochemically silent [9]. *SDHx* mutation carriers can therefore be diagnosed preferentially by normetanephrine and 3-methoxytyramine while, in cases of silent phenotypes, circulating chromogranin A may be a useful marker [51,52,53]. It is important to note that there are multiple other diseases, conditions, and medications leading to false positive chromogranin A results. Diseases potentially leading to elevated chromogranin A levels are other endocrine diseases/tumors (pulmonary/gastrointestinal neuroendocrine tumors, medullary thyroid carcinomas, pituitary tumors, hyperthyroidism, hyperparathyroidism, small cell lung cancer, prostate cancer, breast cancer, ovary carcinoma), systemic inflammatory diseases (systemic rheumatoid arthritis, chronic bronchitis, chronic airway obstruction in smokers), renal insufficiency, gastrointestinal disorders (chronic atrophic gastritis, pancreatitis, inflammatory bowel disease, irritable bowel syndrome, liver cirrhosis, chronic hepatitis, hepatocellular carcinoma, pancreatic and colon cancer), cardiovascular diseases (arterial hypertension, cardiac insufficiency, acute coronary syndrome), drugs (proton pump inhibitors, H2 blockers, steroids), and pregnancy. Therefore, it is especially important to instruct the patient to withhold proton pump inhibitors for at least one week, optimally for two to three weeks, prior to assessment of chromogranin A. H2 blockers should be withheld at least two days prior to chromogranin A assessment if possible.

Since biochemical testing may not be applicable in patients with non-functioning PCC/PGL, clinicians have to rely on repeat imaging for surveillance.

Patients with chromaffin cell tumors due to cluster 2 mutations are usually diagnosed by elevations in both normetanephrine and metanephrine or only metanephrine [47,54,55]. 

#### Conclusion/Practical Tips

Measurements of plasma free normetanephrine, metanephrine, and 3-methoxytyramine, or urinary normetanephrine and metanephrine, are the recommended biochemical tests for PCC/PGL screening and follow-up [39,54,56,57,58] (no caffeine, black tea, nicotine, alcohol, bananas, cheese, almonds, nuts, chocolate, eggs, or vanilla three days prior to assessment).

Especially in the case of a history/suspicion of PGLs, metastatic disease or in *SDHx* mutation carriers, additional assessments of plasma 3-methoxytyramine (but not urinary 3-methoxytyramine) are useful [17] since highly elevated 3-methoxytyramine levels may suggest the presence of metastatic tumors [9,17]. Such patients may be triaged for pre-operative staging (if possible by radionuclide imaging, see below) [39]. 

In the case of a suspected or diagnosed non-functional PGL/PCC, chromogranin A should be measured (no proton pump inhibitor at least one week prior to assessment).

### 3.2. Imaging for Diagnosis, Staging, and Follow-Up

For the localization of adrenal PCCs, anatomic imaging with magnetic resonance imaging (MRI) or computed tomography (CT) has almost 100% sensitivity [42,59,60]. However, for *SDHx* mutated PGLs, HN PGLs, metastatic disease, and small, multiple PCCs/PGLs, both CT/MRI and meta-[^123^I]iodobenzylguanidine ([^123^I]MIBG) single-photon emission computed tomography (SPECT) suffer from significantly lower sensitivity compared to more recent radionuclide imaging [59,61,62]. 

A recent meta-analysis of pooled PCC/PGL detection by radionuclide imaging showed the highest sensitivity (93%) for ^68^Gallium-labeled somatostatin receptor analogs (SSAs) positron emission tomography/computed tomography ([^68^Ga]Ga-DOTA-SSA PET/CT), the second highest sensitivity for dihydroxy-[^18^F]fluorophenylalanine ([^18^F]FDOPA) PET/CT (80%), and the lowest sensitivity for [^18^F]fluorodeoxyglucose ([^18^F]FDG) PET/CT (74%) [63]. 

However, in order to choose the imaging modality with the highest sensitivity, it is important to consider that different sub-groups of PCC/PGL patients show different sensitivities to [^68^Ga]Ga-DOTA-SSAs, [^18^F]FDOPA, and [^18^F]FDG PET/CT, respectively. The sensitivities to the different imaging modalities depend on the presence of PGL versus PCC, HN PGLs, sporadic metastatic, or non-metastatic disease, cluster 1 Krebs cycle-related *SDHx* mutations and *VHL/EPAS1* pseudohypoxia-related mutations or cluster 2 kinase signaling-associated mutations (Table 2) [64].

Highest lesion-based diagnostic sensitivity of [^68^Ga]Ga-DOTA-SSA PET/CT has been found for the following disease characteristics (Table 2):

(1) Metastatic *SDHB*-related PCCs/PGLs ([^68^Ga]Ga-DOTA-SSA 99% vs. [^18^F]FDOPA 61% vs. [^18^F]FDG PET/CT 86%) [61];
(2) *SDHA*-related PCCs/PGLs (highest lesion detection sensitivity: [^68^Ga]Ga-DOTA-SSA > [^18^F]FDG > [^18^F]FDOPA PET/CT) [65];
(3) *SDHD*-related PCCs/PGLs ([^68^Ga]Ga-DOTA-SSA 99% vs. [^18^F]FDOPA 87% vs. [^18^F]FDG PET/CT 62%) [66];
(4) Pediatric *SDHx*-related PGLs/PCCs ([^68^Ga]Ga-DOTA-SSA 94% vs. [^18^F]FDG PET/CT 79%) [67];
(5) HN PGLs (frequently *SDHD*-related) ([^68^Ga]Ga-DOTA-SSA 100% vs. [^18^F]FDOPA 97% vs. [^18^F]FDG PET/CT 71%) [68,69];
(6) PGLs [pooled lesion-based sensitivity of [^68^Ga]Ga-DOTA-SSA PET/CT was higher, compared to [^18^F]FDOPA PET/CT in two different studies ([^68^Ga]Ga-DOTA-SSA 99% and 100% vs. [^18^F]FDOPA PET/CT 68% and 71%, respectively)] [69,70];
(7) Sporadic metastatic PCCs/PGLs ([^68^Ga]Ga-DOTA-SSA 98% vs. [^18^F]FDOPA 75% vs. [^18^F]FDG PET/CT 49%) [71].

A higher lesion-based sensitivity of [^18^F]FDOPA, compared to [^68^Ga]Ga-DOTA-SSA, has been shown for the following disease characteristics (Table 2):

(1) (Sporadic) PCCs ([^18^F]FDOPA 94% vs. [^68^Ga]Ga-DOTA-SSA PET/CT 81%) [69];
(2) Moreover, for *EPAS1 (HIF2A)*-, *PHD1/2-*, *FH-*, and *MAX*-related PGLs, [^18^F]FDOPA PET/CT seems to be the imaging modality with the highest lesion-based sensitivity (for *MAX*-related PCCs/PGLs [^18^F]FDOPA 91% vs. [^68^Ga]Ga-DOTA-SSA 57% vs. [^18^F]FDG PET/CT 18%) although these findings have to be confirmed in a larger patient group with *FH* mutations [72,73,74,75];
(3) *VHL*-related PGLs/PCCs belong to the pseudohypoxia group without leading to succinate accumulation and show high [^18^F]FDOPA uptake but variable [^18^F]FDG uptake which probably makes [^18^F]FDOPA PET/CT the most sensitive imaging modality in *VHL*-related PCCs/PGLs;
(4) In the kinase signaling group, [^18^F]FDOPA also seems to be the most sensitive radiopharmaceutical due to high uptake by the tumor and low uptake in the remaining adrenal gland; however, a head-to-head comparison with [^68^Ga]Ga-DOTA-SSA and [^18^F]FDG PET/CT has only been performed for *MAX*-related PCCs/PGLs as yet [74].

#### Conclusion/Practical Tips 

For PCC screening in the case of elevated metanephrines, CT (or MRI in young patients) should usually be sufficient. 

However, in *all* patients with PCC/PGL (except for patients with low risk of metastatic disease, i.e., epinephrine secreting PCC <3–5 cm), whole-body CT or MRI or radionuclide imaging is recommended prior to surgery in order to rule out metastatic disease/multiplicity which may affect the decision regarding surgery [39]. 

For HN PGLs, sporadic sympathetic PGLs, metastatic tumors, and *SDHx*-related PCCs/PGLs [76], [^68^Ga]Ga-DOTA-SSA PET/CT should be the first choice radionuclide imaging for diagnosis, staging, and follow-up (Table 1 and Table 2). Another advantage of [^68^Ga]Ga-DOTA-SSA PET/CT is its predictive power for the efficacy of peptide receptor radionuclide therapy (PRRT) (see below). If [^68^Ga]Ga-DOTA-SSA PET/CT is not available, for *SDHx*-related PCCs/PGLs, [^18^F]FDG PET/CT should be the second choice; however, for non-*SDHx*-related PGLs and (*SDHD*-related) HN-PGLs [^18^F]FDOPA should be the second choice (Table 2).

In contrast, for (sporadic) PCCs, *VHL, HIF2A*, and kinase signaling-associated (*RET, NF1, MAX*) PCCs/PGLs [^18^F]FDOPA PET/CT should be the first choice radionuclide imaging for diagnosis, staging, and follow-up (Table 1 and Table 2); if not available [^68^Ga]Ga-DOTA-SSA PET/CT should be the second choice except for *HIF2A* and *PHD1/2.* For *HIF2A* and *PHD1/2* [^18^F]FDG PET/CT should be the preferred second choice (Table 2).

### 3.3. Biopsy Is Not Recommended

There is no recommendation for performing biopsy in PCCs/PGLs. The European guideline for adrenal incidentalomas [77] claims that biopsy of an adrenal tumor should only be performed if:

○ There is another extra-adrenal malignancy in the patient’s history;
○ The tumor is non-functioning (especially non-functioning PCCs/PGLs);
○ Not judged as benign on imaging;
○ Biopsy would change patient management.

Therefore, biopsy should only be considered in special cases of non-functioning potential PCCs/PGLs when all biochemistry is negative and it would change patient management, for example, due to another extra-adrenal malignancy in the patient’s history. Otherwise, the adrenal mass should generally be surgically resected due to the risk of a catastrophic complication related to a biopsy of a functioning PCC/PGL [78].

#### Conclusion/Practical Tips 

We strongly discourage image-guided biopsy of any adrenal or suspicious retroperitoneal mass without pre-biopsy biochemical case detection testing.

### 3.4. Immunohistochemistry: Biomarkers

Besides typical morphology, well-known biomarkers for PCC/PGLs are positive immunohistochemistry for chromogranin A, synaptophysin, and S100; however, this does not allow differentiation from any other neuroendocrine tumors. This in turn may be a problem if the patient does not show a typical clinical presentation or biochemistry for a PCC/PGL. In these cases, negativity for keratin and site-specific transcription factors for neuroendocrine tumors as well as positivity for tyrosine hydroxylase (except for non-functioning HN PGLs) and GATA-3 immunohistochemistry seem to be valuable biomarkers to confirm the diagnosis of a PCC/PGL in most cases, as recently reviewed [20]. Future potential relevant biomarkers suggesting metastatic potential may be TERT structural variants [79], BUP1 [80], chromogranin B [81], ERBB2 [82], and/or the well-known SDHB immunohistochemistry [83].

## 4. Follow-Up

It should be emphasized that *all* patients with a history of a PCC/PGL are at risk of recurrence—even after complete (R0) resection—and *any* PCCs/PGL may have metastatic potential [19,39]. This is especially important for those patients with large (≥5–6 cm) tumors [64]. Moreover, extra-adrenal location, a noradrenergic or dopaminergic biochemical phenotype, high chromogranin A levels, young age <20 years, multiplicity, and, most importantly, pseudohypoxic cluster 1-related germline mutations (especially *SDHA/B*), are associated with a higher metastatic risk and an adverse prognosis once metastasis is found [16,17,84,85,86,87,88].

Furthermore, pathologists cannot safely determine from histological findings if a PCC/PGL is ”malignant“ or has ”metastatic potential“ since the existing grading systems [26,27] are of limited predictive power and well-validated biomarkers for metastatic diseases are missing [19,28,29,39]. Thus, for *all* patients with a history of a PCC, follow-up for at least 10 years is suggested [39,42]; for high-risk PCC patients (germline mutation, young age <20 years, large tumor ≥5–6 cm, for *SDHB* carriers tumor size ≥3–3.5 cm) and *all* PGL patients lifelong surveillance is recommended—at least consisting of a yearly clinical investigation and assessment of urinary or plasma metanephrines and 3-methoxytyramine (see above) [39,42,88]. In cases of biochemically-silent PCCs/PGLs, additional imaging every one–two years is suggested [39]. In order to minimize radiation exposure, MRI is the preferred imaging modality for follow-up but it can miss tumors in unusual locations.

All patients at risk for a new PCC/PGL due to a germline mutation should also be offered lifelong surveillance [39,88,89]. However, currently, the details of such lifelong surveillance are unclear—especially if and how often imaging should be performed. Accordingly, due to the strong inter-patient heterogeneity depending on the underlying germline mutations and disease characteristics, Crona et al. suggest a personalized surveillance program using genetic mutations together with disease characteristics (Table 3) [32,42,89].

For *VHL, SDHx,* MEN2, *NF1, TMEM127*, and *MAX*, follow-up recommendations and consensus statements have previously been published [64,89,91,92,93,94].

### 4.1. Conclusion/Practical Tips

In patients with elevated metanephrines prior to PCC/PGL surgery, plasma/urine metanephrines and 3-methoxytyramine (plasma only) should be assessed on pain-free recovery three–six weeks after surgery [39]; in patients with elevated chromogranin A prior to PCC/PGL surgery, chromogranin A should be assessed three–six weeks after surgery [39]; in the rare patients with non-functional PCC/PGL (no tumoral synthesis of catecholamines) or with postoperatively elevated metaneprines or 3-methoxytyramine, imaging should be performed three–four months after presumed complete surgery (if possible with the recommended radionuclide imaging, see above) [39]. 

We suggest especially radionuclide imaging three-four months after presumed complete resection of a metastatic/multiple PCC, *any* PGL, or high-risk (*SDHA/B*) mutation carrier in the case of post-operative abnormal biochemistry or non-functional tumor (Table 3). 

At least 10 years follow-up is recommended for all patients with a history of PCC, but if the tumor was initially ≥5–6 cm, lifelong follow-up is recommended.

Lifelong follow-up is recommended for all high-risk patients (every patient with a germline mutation, PGL, young age <20 years, large tumor size ≥5–6 cm, for *SDHB* carriers tumor size ≥3–3.5 cm, multiplicity/recurrence, noradrenergic/dopaminergic phenotype at the initial presentation, moderately to poorly differentiated PCC according to the GAPP classification system). 

A personalized surveillance program is suggested depending on the underlying germline mutation and disease characteristics (Table 3).

For patients with high-risk mutations (especially *SDHA/B*), we suggest clinical/biochemical control every six months and MRI every one–two years (consider CT for suspected lung involvement or use an alternate approach using CT and MRI).

For completely resected metastatic PGL/PCC, we suggest clinical/biochemical control every six months, MRI six months and 12 months after surgery, then annually. CT can be also used, but with more caution and less frequently since it possesses a radiation risk. An alternate approach using CT and MRI is also an option. Additional radionuclide imaging may be considered every two-three years, especially in the case of high-risk mutations (*SDHA/B*). Other risk factors may apply as well, but their validation may be needed. 

For staging purposes of metastatic disease, we suggest whole body cross-sectional CT or MRI every four–six months and radionuclide imaging every one–two years depending on whether PRRT is considered as a treatment option; a patient’s age and the growth rate/grading of the tumor are also important factors to be considered.

### 4.2. Perspectives

For all above recommendations on follow-up, it should be considered, although it appears logical, that earlier diagnosis of PCC/PGL by follow-up surveillance should provide for better outcomes. This has not been established by any evidence-based study. One recent study provides suggestive evidence for improved outcomes with surveillance in mutation carriers [95], but the retrospective nature of that study and associated uneven matching of populations were confounders that do not allow concrete conclusions about the benefits of follow-up. The international multicenter prospective cohort study PROSPHEO including patients with a history of PCC/PGL, newly-diagnosed PCC/PGL, or mutations in PCC/PGL susceptibility genes from different centers in Germany and Switzerland over 18 years may eventually be able to answer questions concerning benefits of follow-up surveillance as well as the optimal follow-up procedures.

## 5. Therapy

Surgery is always the therapy of choice of non-metastatic PCC/PGL, whenever possible [96,97]. However, surgery of non-functioning HN PGLs has to be carefully balanced against surgery-related morbidities, especially for the cranial nerves for vagal and jugular PGLs [98,99]. In cases of a high risk related to surgery, radiotherapy/radiosurgery (gamma-knife/cyberknife) might be a less invasive option with non-curative but controlling outcomes [100,101,102]. 

In patients with hereditary PCCs, cortical sparing surgery should always be considered since there is frequently a high risk of bilateral PCCs in hereditary disease, and cortical-sparing surgery was not associated with decreased survival despite PCC recurrence in 13% of cases in a very recently published study [103].

With metastatic disease, primary tumor resection should be recommended if feasible in order to alleviate cardiovascular and other symptoms from catecholamine excess or from tumor invasion, and to minimize the target for radiopharmaceutical therapies [31,104,105]. Moreover, several studies have shown that surgical resection of the primary tumor is associated with improved survival even with metastatic disease [104,106,107]. In addition, complete metastatic surgery may be considered in oligo-metastatic PCC/PGL on a case-by-case decision, although there is only little evidence for such an approach from single case reports [108,109]. 

Watchful waiting with frequent follow-up may be the optimal initial approach in patients with non-functioning HN PGL, especially without evidence of significant tumor growth and/or compression of surrounding structures.

Conventional external beam radiation therapy (cEBRT) or radiosurgery (gamma-knife/cyberknife) are well-established methods in the case of bone metastases and also may play a significant palliative role in oligo-metastatic scenarios [102,110].

Minimally-invasive procedures such as radiofrequency ablation, cryoablation, and ethanol injection may be considered in the treatment of metastatic PCC/PGL, especially in oligo-metastatic disease [111,112].

Bisphosphonate or denosumab therapy should be considered in the case of bone metastases by analogy with other types of neuroendocrine tumors.

Adequate blood pressure control with alpha adrenoceptor blockade at least 10–14 days prior to surgery is essential in functioning PCCs/PGLs to prevent severe cardiovascular events during surgery [12,113,114]. In palliative scenarios, alpha adrenoceptor blockade should also be considered—balanced against side effects—to alleviate hormonal symptoms and prevent complications from catecholamine excess [42]. It has been conventional to use phenoxybenzamine at starting doses approximating 10 mg 2–3x per day, although other similar drugs such as doxazosin and prazosin have been used. There is no clear evidence for the superiority of one alpha-blocker for the pre-operative blockade of PCC/PGL patients, as previously reviewed [115]. Nevertheless, perioperative hypertension seems to be slightly better controlled with phenoxybenzamine (especially in those patients with high catecholamine or metanephrine levels), although with more pronounced postoperative hypotension. Indeed, there were fewer side effects in the doxazosin group [115]. Moreover, in functioning metastatic PCCs/PGLs, pre-treatment alpha blockade is recommended prior to initiation of therapy to prevent symptomatic catecholamine release in response to locoregional or systemic treatment. Furthermore, it is important to mention that beta adrenoceptor blockers must not be given prior to initiation of an adequate alpha adrenoceptor blockade [114].

**Figure 1:**
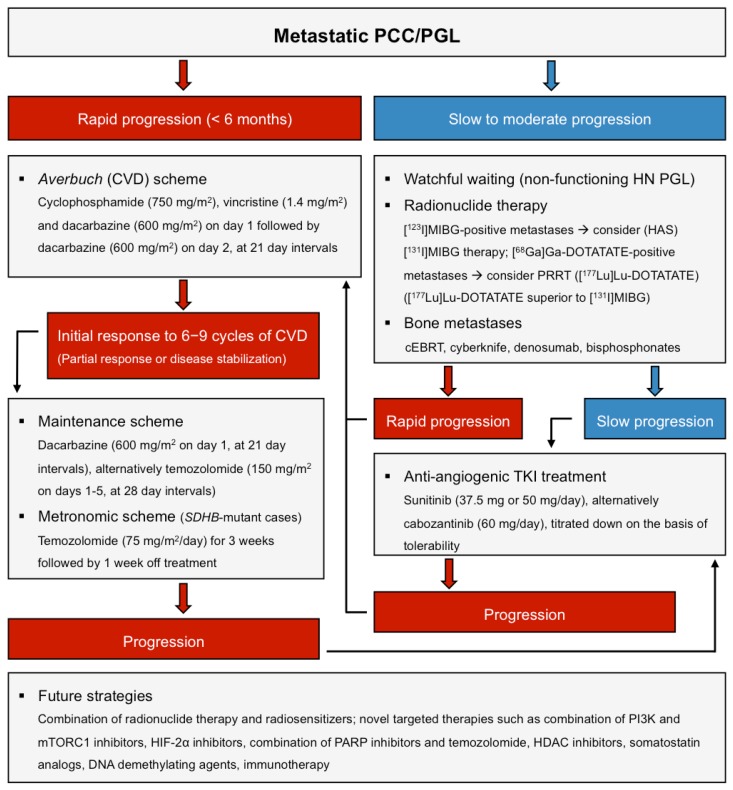
(Modified from [34]): Therapy options in metastatic PCCs/PGLs.

For metastatic PCCs/PGLs, there are few established treatment options but radiotherapy ([^131^I]MIBG therapy, recently PRRT) as well as classic chemotherapy (Averbuch scheme, and temozolomide), and different targeted therapy options, have been extensively used outside of controlled clinical trials (Figure 1, modified from [34]) [34,116].

The only officially (FDA)-approved treatment option in the US is high-specific activity (HSA) [^131^I]MIBG therapy (Ultratrace) [117].

### 5.1. Targeted Endoradionuclide Therapy Using [^131^I]MIBG or Radiolabeled Somatostatin Analogs (PRRT)

The best investigated and well established therapy for metastatic PGL/PCC is [^131^I]MIBG therapy, which is preferentially recommended for slow-growing [^123/131^I]MIBG positive metastatic PCCs/PGLs [32,34], although the studies are very heterogeneous and most are retrospective [118]. In a meta-analysis of 17 studies with 243 PCC/PGL patients on [^131^I]MIBG therapy, there was a complete response in 3% of patients, a partial response in 27% of patients and stable disease in 52% of patients: 40% of patients showed a partial hormonal response [118]. In two of these studies, the mean progression-free survival (PFS) of [^131^I]MIBG treated patients was 23.1 and 28.5 months, respectively [118]. However, due to the unknown status regarding tumor progression prior to therapy in most of these studies, no conclusion can be drawn from the rated stabilization observed in these studies [118]. One study included patients only after progressive disease, but PFS and overall survival (OS) were not reported in this study [119]. Objective responses were mainly observed in patients with soft tissue metastases with a prolonged PFS, but there was no demonstrated impact on survival [118]. 

The most common side effects are anorexia, nausea, vomiting, and hematologic toxicity with grade 3–4 neutropenia in 87% of patients and grade 3–4 thrombocytopenia in 83% [118]. However, in long-term survivors there is a risk of myelodysplastic disorders [120]. One problem with conventional [^131^I]MIBG therapy is the relatively low specific activity of the radiopharmaceutical (most of the MIBG molecules are not ^131^I-labeled) potentially leading to less uptake into the tumor and life-threatening side-effects. Very recently, high-specific activity (HSA) [^131^I]MIBG that consists almost entirely of ^131^I-labeled molecules has been developed [117]. A recent multicentric phase II study led to the FDA-approval of HSA [^131^I]MIBG (Ultratrace, *Azedra*) in the United States [117]: 68 patients received at least one and 50/68 patients received two doses of HSA [^131^I]MIBG. Of the 68 patients who received at least one therapeutic dose of HSA [^131^I]MIBG, 17 (25%) had a durable reduction in baseline anti-hypertensive medication use. Of the 64 patients with evaluable disease, most patients (59/64, 92%) showed partial responses (23%, 15/64) or stable disease (44/64, 69%) as the best objective response. The median OS was 36.7 months (95% confidence interval, 29.9–49.1 months) with 18 months for patients who received one therapeutic dose and 44 months for those who received two therapeutic doses [117]. The most common treatment-related side effects were nausea, myelosuppression, and fatigue [117], with a higher rate of hematologic toxicity compared to conventional [^131^I]MIBG-therapy.

Since most PCCs/PGLs strongly express somatostatin receptor subtype 2 (SSTR2) [121,122], PRRT using radiolabeled somatostatin analogs has been studied in several small studies [123,124,125,126,127,128,129,130,131,132] and recently reviewed [116]. Therefore, latterly, PRRT—a treatment option that has already been approved for gastro-enteropancreatic and pulmonary neuroendocrine tumors in many countries [133]—has also been suggested as an effective treatment option for metastatic PCCs/PGLs [116]. PRRT planning is always preceded by somatostatin receptor imaging as part of a theranostic approach. Although most studies consist of small numbers and limited follow-up, in one direct comparison study the percentage of patients with tumors showing disease stabilization was significantly greater after PRRT using [^177^Lu]Lu-DOTA(Tyr^3^)octreotate (TATE) (100%) compared to [^131^I]MIBG (62.5%), with a longer PFS/OS of 38.5/60.8 months in the [^1^^77^Lu]Lu-DOTATATE group versus 20.6/41.2 months in the [^131^I]MIBG group [127]. The benefit of [^177^Lu]Lu-DOTATATE, compared to [^131^I]MIBG, regarding PFS and OS was even stronger in the subgroup of PGLs (PFS/OS [^177^Lu]Lu-DOTATATE: 22.8/60.8, PFS/OS [^131^I]MIBG: 14.4/38.5) [127]. Further clinically controlled studies (e.g., NCT04029428, NCT03923257, NCT03206060) should be awaited with regard to recommendations and guidelines, including the selection of the radionuclide (^90^Y, ^177^Lu; another potential future option is the use of α-emitting radionuclides [134]), doses and dose regimens, but also the consideration of specific risk constellations (nephrotoxicity, patient age, co-medication with SSA). 

Most common treatment-related side effects of PRRT which is already well-studied in neuroendocrine tumors [133] are renal or hematological (bone marrow) toxicities that can be minimized by adequate precautions and proper and safe dosing [135,136,137,138]. 

In order to maximize radionuclide uptake in the tumor with minimal risk to organs at risk (especially the kidney), an internal patient-specific dosimetry prior to PRRT is a potential future approach (personalized PRRT) [139,140,141,142]. However, currently, a fixed empiric dose is applied in analogy to the NETTER-1 trial in most centers [133] and dosimetry is not a standard procedure.

Interestingly, there are few or no data on the use of unlabeled long-acting somatostatin analogs, such as octreotide LAR or lanreotide autogel, in therapy. These are administered once a month, and in patients with other forms of neuroendocrine tumors (NETs), especially pancreatic and midgut NETs, they cannot only inhibit hormonal secretion but, according to two large-scale trials, attenuate tumor progression [143,144]. As such agents are usually well-tolerated with generally mild adverse effects, they may be trialed in patients with the aim of lowering catecholamine secretion and possibly stabilizing tumor growth, especially when ^68^Ga[Ga]-DOTATATE PET/CT is positive. 

#### Conclusion/Practical Tips 

[^131^I]MIBG therapy is still recommended for slow-growing metastatic PCC/PGL. 

Very recently, HSA [^131^I]MIBG (*Azedra*) has been FDA-approved in the United States for the treatment of metastatic PCC/PGL. However, HSA [^131^I]MIBG was associated with a higher rate of hematologic toxicity although it can result in long-lasting disease stabilization and it may be preferable in patients with good bone marrow reserve. 

One head-to-head comparison study indicates that somatostatin receptor targeted PRRT may be superior to conventional [^131^I]MIBG-therapy regarding treatment response, PFS and OS in metastatic PCC/PGL, especially in the subgroup of PGLs, and may be recommended for slow-growing metastatic PCC/PGL.

### 5.2. Chemotherapy

The second most studied therapy recommended for rapidly progressing metastatic PCC/PGL is conventional chemotherapy with cyclophosphamide, vincristine, and dacarbazine (CVD, Averbuch scheme: Cyclophosphamide 750 mg/m^2^, vincristine 1.4 mg/m^2^, and dacarbazine 600 mg/m^2^ on day 1 and dacarbazine 600 mg/m^2^ on day 2, at 21 day intervals) [145,146]. In a meta-analysis of the largest studies, CVD led to a complete response in 4%, a partial response in 37%, and stable disease in 14% of patients [145]. PFS was only reported in two of these studies with 20 and 40 months, respectively. However, due to missing information regarding tumor progression prior to therapy, no valid conclusions can be drawn from these PFS data [145]. There is only one study solely including patients with progression prior to therapy which showed a radiological and clinical response in 33% of patients [105]. This is also the only study showing a significant survival benefit for patients who responded to CVD chemotherapy: There was a significant effect of response to chemotherapy on median OS (according to a multivariate Cox proportional hazard model analyses) [105]. The median OS of radiological responders was 6.4 years versus 3.7 years for non-responders [105]. The CVD regimen has been shown to be especially effective in *SDHB*-related PCC/PGLs [145,147,148]. 

Prolonged CVD chemotherapy (median of 20.5 cycles) in 12 patients with *SDHB* mutations led to a total response in 83% of patients [partial response 8/12 (66.7%) patients, complete response 2/12 (16.7%) patients, assessed by Response Evaluation Criteria in Solid Tumors (RECIST)] and a PFS/OS of 930 and 1190 days, respectively [148]. On a case-by-case basis, prolonged CVD therapy can be considered, especially for metastatic *SDHB*-related PCC/PGLs. 

Monotherapy with the DNA-alkylating chemotherapeutic temozolomide, an oral metabolite of dacarbazine, showed a partial response (33%) or stable disease (47%) in a total of 80% of patients with *SDHB* mutations, and thus may be used as a single agent treatment, or alternatively could be considered as a maintenance regime for tumor stabilization subsequent to six–nine cycles of CVD chemotherapy (150 mg/m^2^ on days 1–5, at 28 day intervals) [149,150]. Down-regulation of the DNA repairing enzyme O-6-methylguanine-DNA methyltransferase (MGMT) via hypermethylation in *SDHB* mutated tumors appears to lead to increased susceptibility of *SDHB*-related PCCs/PGLs to temozolomide [149,151,152,153]. 

In the case of intolerance to temozolomide monotherapy, a combination of a metronomic scheme with long-term low-dose temozolomide (75 mg/m^2^ per day for three weeks followed by one week off treatment) and high-dose lanreotide autogel (120 mg s.c. every 14 days) may be an alternative in order to stabilize PCC/PGL growth (low MGMT levels seem to be beneficial), as reported for two patients [150]. 

The well-known side effects of CVD chemotherapy include amongst others nausea, vomiting, myelosuppression, peripheral sensory and autonomic neuropathy (vincristine), hemorrhagic cystitis (cyclophosphamide), and infertility [105,154]. 

Whether adjuvant chemotherapy with four–six cycles of CVD after surgery is beneficial, has not as yet been studied. There are no data providing evidence for adjuvant chemotherapy.

#### Conclusion/Practical Tips

CVD chemotherapy (cyclophosphamide 750 mg/m^2^, vincristine 1.4 mg/m^2^, and dacarbazine 600 mg/m^2^ on day 1 and dacarbazine 600 mg/m^2^ on day 2, at 21 day intervals) is recommended for rapidly progressive (<6 months) metastatic PCC/PGL and especially effective in *SDHB*-related disease. 

On a case-by-case decision, prolonged treatment with CVD chemotherapy with 20 cycles of CVD is suggested, especially in the case of patients with *SDHB* mutations.

Alternatively, temozolomide monotherapy (150 mg/m^2^ on days 1–5, at 28 day intervals) or a metronomic scheme with temozolomide (75 mg/m^2^ per day for three weeks followed by one week off treatment) may be considered, either as initial therapy or following stabilization with CVD.

### 5.3. Targeted Therapy and Immunotherapy

Different receptor tyrosine kinase inhibitors (TKIs) (sunitinib, cabozantinib, axitinib, lenvatinib, and pazopanib) are currently under evaluation as treatment options for metastatic PCCs/PGLs. They all have anti-angiogenic effects and may be interesting therapy options for cluster 1 and cluster 2 mutated PCCs/PGLs. 

The best studied TKI is sunitinib, which is already approved by the FDA and by the European Medicines Agency (EMA) for pancreatic neuroendocrine tumors, renal cell cancer, and gastrointestinal stromal tumors. Sunitinib leads to inhibition of VEGF1/2 receptors, platelet-derived growth factor-β receptor (PDGFR) and RET. The largest retrospective study included 17 patients of which 14 were evaluable for tumor responses to sunitinib (dose: 37.5 mg or 50 mg) [155]. Of the 14 patients, a total of 8/14 (57%) of the patients showed a partial response (3/14, 21%) or stable disease (5/14, 36%) [155]. However, the median PFS was only 4.1 months, although there was a much longer PFS in the responders compared to the non-responders. The PFS of the three partial responders was 11, 12, and 4.5 months, respectively [155]. The PFS of three patients with stable disease was 27, 8, and 6 months, respectively, and two other patients with stable disease experienced no progression until the end of the observation period (36 months). One of these patients remained on targeted combination therapy with sunitinib and the mTORC1 inhibitor rapamycin for 1.5 years until the end of the observation period [155]. In the non-responders, the PFS was 0.4–4 months. A total of 6/8 (75%) of the patients with stable disease or partial responses in this study were *SDHB* mutation carriers, indicating clinical benefit especially for *SDHB*-related PCCs/PGLs [155]. 

Importantly, the largest prospective sunitinib phase II multicenter study has recently been published (regime: 50 mg sunitinib daily for four weeks, followed by two weeks off treatment corresponding to one cycle): The total disease control rate (stable disease or partial response) was 83% (95% CI: 61–95%): 3/23 (13%) patients showed a partial response. All responders were carriers of germline mutations (*SDHA, SDHB, RET*). The median PFS was 13.4 (95% CI: 5.3–24.6) months [156]. The *RET*-mutated patient with MEN2A was still on sunitinib therapy by the time the study was published (after 64 cycles) which may implicate that *RET* and *SDHx* mutation carriers as benefiting the most [156]. 

At present, sunitinib is being investigated in the first randomized placebo-controlled phase II clinical trial in advanced PCC/PGL (FIRST-MAPP, NCT01371202), recruitment (n = 74) has been completed and results are pending. 

Common side effects of sunitinib are fatigue, nausea, vomiting, diarrhea, taste changes, heartburn, severe hypertension, and myelosuppression.

Another TKI, the c-Met inhibitor cabozantinib, which was more effective than sunitinib in renal cell carcinoma and in human PCC/PGL primary culture [157,158,159], is currently being investigated in a phase II clinical study in 11 metastatic PCC/PGL patients (initial dose: 60 mg, titrated down on the basis of tolerability) (NCT02302833). Preliminary data have shown tumor size reduction and disease stabilization in most patients, with a median PFS of 11.2 months [160]. The side effects of cabozantinib are similar to those of sunitinib.

The TKI axitinib (AG-013736) is currently being investigated in a phase II non-randomized clinical trial including 14 patients with metatstatic PCC/PGL (NCT01967576). Moreover, another phase II clinical trial is evaluating the efficacy of the TKI lenvatinib, inhibiting VEGFR1/2/3, in advanced PCC/PGL (NCT03008369). The pazopanib trial had to be terminated due to gastrointestinal and severe cardiovascular events [161].

Treatment with the mTORC1 inhibitor everolimus led to disease stabilization in five out of seven patients with advanced PCC/PGL in a small phase II study (NCT01152827) [162], but another study showed no effect of the agent on its own [163]. However, one patient treated with 25 mg sunitinib in combination with 4 mg of the mTORC1 inhibitor rapamycin (as mentioned above) experienced maintained long-term disease control [155]. Accordingly, it is possible that targeted therapies in combination may be more effective at lower doses compared to single treatment approaches. 

Consistently, we have already shown in several in vitro and in vivo studies that targeted drug combinations show synergistic anti-tumor effects in PCCs/PGLs [159,164,165,166,167]. Moreover, very recently, we established a method to screen multiple targeted drug combinations—some are already in use for other types of cancers—ex vivo in human PCC/PGL primary cultures of individual patient tumors [159]. These data may then be correlated to the signaling pathway alterations and the individual genetic background of the tumor [159]. This will hopefully pave the way to customized combination therapy to target individual patient tumors.

Potentially interesting novel targeted therapy approaches for PCCs/PGLs (especially cluster 1-related) include HIF-2α inhibitors, inhibitors of the DNA repairing enzyme Poly(ADP-ribose) polymerase (PARP) (especially in combination with temozolomide), histone deacetylase (HDAC) inhibitors, SSTR2 analogs (see above), or DNA demethylating agents (DNA methyltransferase inhibitor SGI-110 is under investigation in a phase II clinical trial for treatment of *SDHx*-related PCCs/PGLs, NCT03165721), as recently reviewed [34,168].

Pseudo-hypoxia may prevent the immune system from recognizing cluster 1-related PCCs/PGLs through inactivation of cytotoxic T-cell lymphocytes, activation of immune-suppressive monocytes and increased expression of the immune checkpoint protein programmed death-ligand 1 (PD-L1) and its receptor [169,170,171]. Therefore, immunotherapy is currently being studied in advanced PCC/PGL in two different phase II clinical studies [nivolumab plus ipilimumab (NCT02834013) and pembrolizumab (NCT02721732), respectively].

#### Conclusion/Practical Tips 

In the case of progression after chemotherapy or radionuclide therapy, or if chemotherapy or radionuclide therapy are not possible/tolerated by the patient, the TKIs sunitinib (37.5 mg or 50 mg daily) or cabozantinib (60 mg daily, or titrated down to a tolerable dose) may be considered, especially for *RET* and *SDHx*-mutation carriers. 

Several targeted therapies (PI3K inhibitors in combination with mTORC1 inhibitors, HIF-2α inhibitors, PARP inhibitors, SSTR2 analogs, HDAC inhibitors, DNA demethylating agents) and immunotherapy are currently under investigation and may have strong potential for future personalized therapy approaches. 

## 6. Outlook 

Customized combination therapy (targeted therapy combinations [159], combinations of targeted therapy with immunotherapy, or targeted therapy combined with PRRT) to target individual patient tumors depending on their underlying germline/somatic mutation and disease characteristics, are likely to be the future directions of therapeutic options for these fascinating but complex tumors.

## Figures and Tables

**Table 1:** Pheochromocytomas and paragangliomas (PCC/PGL) characteristics depending on underlying germline/somatic mutations and their clinical presentation and evaluation.

Genes	*Cluster 1 (Pseudohypoxic Krebs Cycle-Related): SDHx (SDHA, B, C, D, F2), FH, MDH2 (10–15% of PCC/PGL)*	*Cluster 1 (Pseudohypoxia VHL/EPAS1-Related: VHL*, *EPAS1/2* (*HIF2A), PHD1/2 (15–20% of PCC/PGL)*	*Cluster 2 (Kinase Signaling-Related): RET, NF1, MAX, TMEM127, HRAS (50–60% of PCC/PGL)*	*Cluster 3 (Wnt Signaling-Related): CSDE1, MAML3 5–10% of PCC/PGL)*
Percentage of germline mutations [2]	Germline 100%	Germline 25%	Germline 20%	Germline 0%
Signaling pathways	Pseudohypoxia, Krebs cycle-related, HIF-2α stabilization and signaling	Pseudohypoxia, *VHL/EPAS1*-related signaling	Kinase signaling: PI3K/AKT, RAS/RAF/ERK, mTORC1/p70S6K	Wnt signaling
Biochemistry	Normetanephrine, 3-methoxytyramine	Normetanephrine	Normetanephrine and metanephrine or metanephrine alone	Normetanephrine, metanephrine
Imaging	[^68^Ga]Ga-DOTA-SSA PET/CT (possibly except for *FH*)	[^18^F]FDOPA PET/CT (possibly also for *FH*)	[^18^F]FDOPA PET/CT in most	Unknown
Tumor location	Mostly extra-adrenal	Adrenal, extra-adrenal	Adrenal	Unknown
Metastatic risk	High-Intermediate	Intermediate-Low	Low	Intermediate
Age of presentation	Early (under 20–30 year-old)	Early, some often during childhood	Late but some can present early	Unknown

**Table 2:** Choice of radionuclide imaging based on the underlying germline/somatic mutations and disease characteristics, modified from Taieb et al. [64].

Radionuclide Imaging Method for Diagnosis, Staging, and Follow-Up	(Sporadic) PGL, Multifocal/Metastatic Disease, *SDHx*	(Sporadic) PCC (Except *SDHx*): *VHL, EPAS1* (*HIF2A*), *FH*, *PHD1/2*, Kinase Signaling-Associated (*NF1, RET, MAX*)
**First choice**	[^68^Ga]Ga-DOTA-SSA PET/CT reveals the predictive power for efficacy of PRRT	[^18^F]FDOPA PET/CT
**Second choice**	[^18^F]FDG PET/CT for *SDHx*-related PGL (except for *SDHD*-related HN PGL)	[^68^Ga]Ga-DOTA-SSA PET/CT (except for *EPAS1* (*HIF2A*), *PHD1/2*)
**Second choice**	[^18^F]FDOPA PET/CT for *SDHD*-related HN PGL	[^18^F]FDG PET/CT for *EPAS1/2* (*HIF2A*), *PHD1/2*

**Table 3:** Follow-up depending on the underlying germline mutation and disease characteristics (after presumably curative surgery).

Follow-Up	High Risk Group: Completely Resected Metastatic PCC/PGL, Completely Resected Non-Metastatic Sympathetic PGL, *SDHA*, *SDHB*, *SDHD* (Except for HN PGL), *EPAS1 (HIF2A)*, *PHD1/2*	Intermediate Risk Group: Completely Resected HN PGL, Completely Resected High-Risk PCC, *SDHAF2, SDHC, VHL Type 2, FH* (Data Is Limited), *TMEM127, MAX, RET* (High Risk Allele), Recurrence, Multiplicity	Low Risk Group: Completely Resected Low-Risk PCC, *VHL* Type 1, *NF1, RET* (Low to Moderate Risk Allele)
**Clinical/metanephrines**	6–12 months	12 months	12–24 months
Imaging (preferably MRI *, less frequently CT *, but an alternate approach is allowed and by some recommended)	12–24 months (shorter 12-months interval may be based on metastases, the initial size of a tumor, secretory pattern, and young age), consider CT, especially for lung involvement	24–36 months (36 months applies for small sporadic metanephrine secreting tumors)	Optional at screening
**Special cases**	Completely resected metastatic PGL/PCC, *SDHA/B* PCC/PGL, sympathetic PGL: May consider radionuclide imaging 3–4 months after surgery if abnormal biochemistry or non-functioning PCC/PGL Completely resected metastatic PGL/metastatic PCC (independent of biochemistry): MRI 6 months, 12 months after surgery, then MRI yearly, consider specific radionuclide imaging every 24–36 months		

Red letters: Especially high risk; definition of high-risk PCC: Diameter ≥5–6 cm, or ≥3–3.5 cm for *SDHB* carriers, or young age <20 at diagnosis, or moderately to poorly differentiated PCC according to GAPP classification system, norepinephrine/dopamine hyper-secretion; RET low risk alleles (incidence around 10%): G533C, K666E, L790F, V804L, V804M, S891A; RET moderate risk alleles (incidence 20–30%): C609F/G/R/S/Y, C611F/G/S/Y/W, C618F/R/S, C620F/R/S, C630R/Y; RET high risk alleles (incidence around 50%): M918T, D631Y, C634F/G/R/S/W/Y, A883F [90]; RET low and moderate risk alleles: Begin screening for PCC at the age of 16 years; RET high risk alleles: Begin screening for PCC at the age of 11 years (for all *RET* mutations consider medullary thyroid carcinoma) [90]. * Whole body MRI/CT (from skull base to pelvis) in the high risk group, consider focused MRI/CT in the intermediate and low risk group (for example no head and neck imaging in *MAX, FH, NF1*).

## References

[1] Beard C.M., Sheps S.G., Kurland L.T., Carney J.A., Lie J.T. (1983). Occurrence of pheochromocytoma in Rochester, Minnesota, 1950 through 1979. Mayo Clin. Proc..

[2] Fishbein L., Leshchiner I., Walter V., Danilova L., Robertson A.G., Johnson A.R., Lichtenberg T.M., Murray B.A., Ghayee H.K., Else T. (2017). Comprehensive molecular characterization of pheochromocytoma and paraganglioma. Cancer Cell.

[3] Burnichon N., Vescovo L., Amar L., Libe R., De R.A., Venisse A., Jouanno E., Laurendeau I., Parfait B., Bertherat J. (2011). Integrative genomic analysis reveals somatic mutations in pheochromocytoma and paraganglioma. Hum. Mol. Genet..

[4] Sutton M.G., Sheps S.G., Lie J.T. (1981). Prevalence of clinically unsuspected pheochromocytoma. Review of a 50-year autopsy series. Mayo Clin. Proc..

[5] Pamporaki C., Hamplova B., Peitzsch M., Prejbisz A., Beuschlein F., Timmers H., Fassnacht M., Klink B., Lodish M., Stratakis C.A. (2017). Characteristics of pediatric vs adult pheochromocytomas and paragangliomas. J. Clin. Endocrinol. Metab..

[6] Eisenhofer G., Prejbisz A., Peitzsch M., Pamporaki C., Masjkur J., Rogowski-Lehmann N., Langton K., Tsourdi E., Peczkowska M., Fliedner S. (2018). Biochemical diagnosis of chromaffin cell tumors in patients at high and low risk of disease: Plasma versus urinary free or deconjugated O-methylated catecholamine metabolites. Clin. Chem..

[7] Geroula A., Deutschbein T., Langton K., Masjkur J.R., Pamporaki C., Peitzsch M., Fliedner S., Timmers H.J., Bornstein S.R., Beuschlein F. (2019). Pheochromocytoma and paraganglioma: Clinical feature based disease probability in relation to catecholamine biochemistry and reason for disease suspicion. Eur. J. Endocrinol..

[8] Eisenhofer G., Klink B., Richter S., Lenders J.W., Robledo M. (2017). Metabologenomics of phaeochromocytoma and paraganglioma: An integrated approach for personalised biochemical and genetic testing. Clin. Biochem. Rev..

[9] Timmers H.J., Pacak K., Huynh T.T., Abu-Asab M., Tsokos M., Merino M.J., Baysal B.E., Adams K.T., Eisenhofer G. (2008). Biochemically silent abdominal paragangliomas in patients with mutations in the succinate dehydrogenase subunit B gene. J. Clin. Endocrinol. Metab..

[10] Remine W.H., Chong G.C., Van Heerden J.A., Sheps S.G., Harrison E.G. (1974). Current management of pheochromocytoma. Ann. Surg..

[11] Proye C.A., Vix M., Jansson S., Tisell L.E., Dralle H., Hiller W. (1994). “The” pheochromocytoma: A benign, intra-adrenal, hypertensive, sporadic unilateral tumor. Does it exist?. World J. Surg..

[12] Goldstein R.E., O’Neill J.A., Holcomb G.W., Morgan W.M., Neblett W.W., Oates J.A., Brown N., Nadeau J., Smith B., Page D.L. (1999). Clinical experience over 48 years with pheochromocytoma. Ann. Surg..

[13] Mannelli M., Ianni L., Cilotti A., Conti A. (1999). Pheochromocytoma in Italy: A multicentric retrospective study. Eur. J. Endocrinol..

[14] John H., Ziegler W.H., Hauri D., Jaeger P. (1999). Pheochromocytomas: Can malignant potential be predicted?. Urology.

[15] Elder E.E., Skog A.L.H., Hoog A., Hamberger B. (2003). The management of benign and malignant pheochromocytoma and abdominal paraganglioma. Eur. J. Surg. Oncol..

[16] Amar L., Servais A., Gimenez-Roqueplo A.P., Zinzindohoue F., Chatellier G., Plouin P.F. (2005). Year of diagnosis, features at presentation, and risk of recurrence in patients with pheochromocytoma or secreting paraganglioma. J. Clin. Endocrinol. Metab..

[17] Eisenhofer G., Lenders J.W., Siegert G., Bornstein S.R., Friberg P., Milosevic D., Mannelli M., Linehan W.M., Adams K., Timmers H.J. (2012). Plasma methoxytyramine: A novel biomarker of metastatic pheochromocytoma and paraganglioma in relation to established risk factors of tumour size, location and SDHB mutation status. Eur J. Cancer.

[18] Hamidi O., Young W.F., Gruber L., Smestad J., Yan Q., Ponce O.J., Prokop L., Murad M.H., Bancos I. (2017). Outcomes of patients with metastatic phaeochromocytoma and paraganglioma: A systematic review and meta-analysis. Clin. Endocrinol..

[19] Lam A.K. (2017). Update on adrenal tumours in 2017 World Health Organization (WHO) of endocrine tumours. Endocr. Pathol..

[20] Asa S.L., Ezzat S., Mete O. (2018). The diagnosis and clinical significance of paragangliomas in unusual locations. J. Clin. Med..

[21] Koh P.S., Koong J.K., Westerhout C.J., Yoong B.K. (2013). Education and imaging. Hepatobiliary and pancreatic: A huge liver paraganglioma. J. Gastroenterol. Hepatol..

[22] Liao W., Ding Z.Y., Zhang B., Chen L., Li G.X., Wu J.J., Zhang B., Chen X.P., Zhu P. (2018). Primary functioning hepatic paraganglioma mimicking hepatocellular carcinoma: A case report and literature review. Medicine.

[23] Gucer H., Mete O. (2014). Endobronchial gangliocytic paraganglioma: Not all keratin-positive endobronchial neuroendocrine neoplasms are pulmonary carcinoids. Endocr. Pathol..

[24] Huang X., Liang Q.L., Jiang L., Liu Q.L., Ou W.T., Li D.H., Zhang H.J., Yuan G.L. (2015). Primary pulmonary paraganglioma: A case report and review of literature. Medicine.

[25] Fiorentino G., Annunziata A., de Rosa N. (2015). Primary paraganglioma of the lung: A case report. J. Med. Case Rep..

[26] Thompson L.D. (2002). Pheochromocytoma of the Adrenal gland Scaled Score (PASS) to separate benign from malignant neoplasms: A clinicopathologic and immunophenotypic study of 100 cases. Am J. Surg. Pathol..

[27] Kimura N., Takayanagi R., Takizawa N., Itagaki E., Katabami T., Kakoi N., Rakugi H., Ikeda Y., Tanabe A., Nigawara T. (2014). Pathological grading for predicting metastasis in phaeochromocytoma and paraganglioma. Endocr. Relat. Cancer.

[28] Kimura N., Takekoshi K., Naruse M. (2018). Risk stratification on pheochromocytoma and paraganglioma from laboratory and clinical medicine. J. Clin. Med..

[29] Stenman A., Zedenius J., Juhlin C.C. (2019). The value of histological algorithms to predict the malignancy potential of pheochromocytomas and abdominal paragangliomas—A meta-analysis and systematic review of the literature. Cancers.

[30] Stenman A., Zedenius J., Juhlin C.C. (2019). Retrospective application of the pathologic tumor-node-metastasis classification system for pheochromocytoma and abdominal paraganglioma in a well characterized cohort with long-term follow-up. Surgery.

[31] Roman-Gonzalez A., Jimenez C. (2017). Malignant pheochromocytoma-paraganglioma: Pathogenesis, TNM staging, and current clinical trials. Curr. Opin. Endocrinol. Diabetes Obes..

[32] Crona J., Taieb D., Pacak K. (2017). New perspectives on pheochromocytoma and paraganglioma: Toward a molecular classification. Endocr. Rev..

[33] Jochmanova I., Pacak K. (2018). Genomic landscape of pheochromocytoma and paraganglioma. Trends Cancer.

[34] Nölting S., Grossman A., Pacak K. (2018). Metastatic Phaeochromocytoma: Spinning towards more promising treatment options. Exp. Clin. Endocrinol. Diabetes.

[35] Jochmanova I., Yang C., Zhuang Z., Pacak K. (2013). Hypoxia-inducible factor signaling in pheochromocytoma: Turning the rudder in the right direction. J. Natl. Cancer Inst..

[36] Tella S.H., Taieb D., Pacak K. (2017). HIF-2alpha: Achilles’ heel of pseudohypoxic subtype paraganglioma and other related conditions. Eur. J. Cancer.

[37] Timmers H.J., Kozupa A., Eisenhofer G., Raygada M., Adams K.T., Solis D., Lenders J.W., Pacak K. (2007). Clinical presentations, biochemical phenotypes, and genotype-phenotype correlations in patients with succinate dehydrogenase subunit B-associated pheochromocytomas and paragangliomas. J. Clin. Endocrinol. Metab..

[38] Qin N., de Cubas A.A., Garcia-Martin R., Richter S., Peitzsch M., Menschikowski M., Lenders J.W., Timmers H.J., Mannelli M., Opocher G. (2014). Opposing effects of HIF1α and HIF2α on chromaffin cell phenotypic features and tumor cell proliferation: Insights from MYC-associated factor X. Int. J. Cancer.

[39] Plouin P.F., Amar L., Dekkers O.M., Fassnacht M., Gimenez-Roqueplo A.P., Lenders J.W., Lussey-Lepoutre C., Steichen O., Guideline Working Group (2016). European Society of Endocrinology Clinical Practice Guideline for long-term follow-up of patients operated on for a phaeochromocytoma or a paraganglioma. Eur. J. Endocrinol..

[40] Haugen B.R., Alexander E.K., Bible K.C., Doherty G.M., Mandel S.J., Nikiforov Y.E., Pacini F., Randolph G.W., Sawka A.M., Schlumberger M. (2016). 2015 American Thyroid Association management guidelines for adult patients with thyroid nodules and differentiated thyroid cancer: The American Thyroid Association guidelines task force on thyroid nodules and differentiated thyroid cancer. Thyroid.

[41] Hageman J.C., Pegues D.A., Jepson C., Bell R.L., Guinan M., Ward K.W., Cohen M.D., Hindler J.A., Tenover F.C., McAllister S.K. (2001). Vancomycin-intermediate Staphylococcus aureus in a home health-care patient. Emerg. Infect. Dis..

[42] Lenders J.W., Duh Q.Y., Eisenhofer G., Gimenez-Roqueplo A.P., Grebe S.K., Murad M.H., Naruse M., Pacak K., Young W.F., Endocrine S. (2014). Pheochromocytoma and paraganglioma: An endocrine society clinical practice guideline. J. Clin. Endocrinol. Metab..

[43] Darr R., Pamporaki C., Peitzsch M., Miehle K., Prejbisz A., Peczkowska M., Weismann D., Beuschlein F., Sinnott R., Bornstein S.R. (2014). Biochemical diagnosis of phaeochromocytoma using plasma-free normetanephrine, metanephrine and methoxytyramine: Importance of supine sampling under fasting conditions. Clin. Endocrinol..

[44] Weismann D., Peitzsch M., Raida A., Prejbisz A., Gosk M., Riester A., Willenberg H.S., Klemm R., Manz G., Deutschbein T. (2015). Measurements of plasma metanephrines by immunoassay vs liquid chromatography with tandem mass spectrometry for diagnosis of pheochromocytoma. Eur. J. Endocrinol..

[45] Boyd J., Leung A.A., Sadrzadeh H., Pamporaki C., Pacak K., Deutschbein T., Fliedner S., Kline G.A. (2019). A high rate of modestly elevated plasma normetanephrine in a population referred for suspected PPGL when measured in a seated position. Eur. J. Endocrinol..

[46] Olson S.W., Yoon S., Baker T., Prince L.K., Oliver D., Abbott K.C. (2016). Longitudinal plasma metanephrines preceding pheochromocytoma diagnosis: A retrospective case-control serum repository study. Eur. J. Endocrinol..

[47] Eisenhofer G., Huynh T.T., Pacak K., Brouwers F.M., Walther M.M., Linehan W.M., Munson P.J., Mannelli M., Goldstein D.S., Elkahloun A.G. (2004). Distinct gene expression profiles in norepinephrine-and epinephrine-producing hereditary and sporadic pheochromocytomas: Activation of hypoxia-driven angiogenic pathways in von Hippel-Lindau syndrome. Endocr. Relat. Cancer.

[48] Sue M., Martucci V., Frey F., Lenders J.M., Timmers H.J., Peczkowska M., Prejbisz A., Swantje B., Bornstein S.R., Arlt W. (2015). Lack of utility of SDHB mutation testing in adrenergic metastatic phaeochromocytoma. Eur. J. Endocrinol..

[49] Eisenhofer G., Lenders J.W., Timmers H., Mannelli M., Grebe S.K., Hofbauer L.C., Bornstein S.R., Tiebel O., Adams K., Bratslavsky G. (2011). Measurements of plasma methoxytyramine, normetanephrine, and metanephrine as discriminators of different hereditary forms of pheochromocytoma. Clin. Chem..

[50] Feldman J.M., Blalock J.A., Zern R.T., Shelburne J.D., Gaede J.T., Farrell R.E., Wells S.A. (1979). Deficiency of dopamine-β-hydroxylase: A new mechanism for normotensive pheochromocytomas. Am. J. Clin. Pathol..

[51] Zuber S., Wesley R., Prodanov T., Eisenhofer G., Pacak K., Kantorovich V. (2014). Clinical utility of chromogranin A in SDHx-related paragangliomas. Eur. J. Clin. Investig..

[52] Hsiao R.J., Parmer R.J., Takiyyuddin M.A., O’Connor D.T. (1991). Chromogranin A storage and secretion: Sensitivity and specificity for the diagnosis of pheochromocytoma. Medicine.

[53] Bilek R., Vlcek P., Safarik L., Michalsky D., Novak K., Duskova J., Vaclavikova E., Widimsky J., Zelinka T. (2019). Chromogranin A in the laboratory diagnosis of pheochromocytoma and paraganglioma. Cancers.

[54] Pacak K., Ilias I., Adams K.T., Eisenhofer G. (2005). Biochemical diagnosis, localization and management of pheochromocytoma: Focus on multiple endocrine neoplasia type 2 in relation to other hereditary syndromes and sporadic forms of the tumour. J. Intern. Med..

[55] Eisenhofer G., Pacak K., Huynh T.T., Qin N., Bratslavsky G., Linehan W.M., Mannelli M., Friberg P., Grebe S.K., Timmers H.J. (2011). Catecholamine metabolomic and secretory phenotypes in phaeochromocytoma. Endocr. Relat. Cancer.

[56] Weise M., Merke D.P., Pacak K., Walther M.M., Eisenhofer G. (2002). Utility of plasma free metanephrines for detecting childhood pheochromocytoma. J. Clin. Endocrinol. Metab..

[57] Lenders J.W., Pacak K., Walther M.M., Linehan W.M., Mannelli M., Friberg P., Keiser H.R., Goldstein D.S., Eisenhofer G. (2002). Biochemical diagnosis of pheochromocytoma: Which test is best?. JAMA.

[58] Eisenhofer G., Lenders J.W., Linehan W.M., Walther M.M., Goldstein D.S., Keiser H.R. (1999). Plasma normetanephrine and metanephrine for detecting pheochromocytoma in von Hippel-Lindau disease and multiple endocrine neoplasia type 2. N. Engl. J. Med..

[59] Jalil N.D., Pattou F.N., Combemale F., Chapuis Y., Henry J.F., Peix J.L., Proye C.A. (1998). Effectiveness and limits of preoperative imaging studies for the localisation of pheochromocytomas and paragangliomas: A review of 282 cases. French Association of Surgery (AFC), and The French Association of Endocrine Surgeons (AFCE). Eur. J. Surg..

[60] Ganguly A., Henry D.P., Yune H.Y., Pratt J.H., Grim C.E., Donohue J.P., Weinberger M.H. (1979). Diagnosis and localization of pheochromocytoma: Detection by measurement of urinary norepinephrine excretion during sleep, plasma norepinephrine concentration and computerized axial tomography (CT-scan). Am. J. Med..

[61] Janssen I., Blanchet E.M., Adams K., Chen C.C., Millo C.M., Herscovitch P., Taieb D., Kebebew E., Lehnert H., Fojo A.T. (2015). Superiority of [^68^Ga]-DOTATATE PET/CT to other functional imaging modalities in the localization of SDHB-associated metastatic pheochromocytoma and paraganglioma. Clin. Cancer Res..

[62] Timmers H.J., Chen C.C., Carrasquillo J.A., Whatley M., Ling A., Eisenhofer G., King K.S., Rao J.U., Wesley R.A., Adams K.T. (2012). Staging and functional characterization of pheochromocytoma and paraganglioma by ^18^F-fluorodeoxyglucose (^18^F-FDG) positron emission tomography. J. Natl. Cancer Inst..

[63] Han S., Suh C.H., Woo S., Kim Y.J., Lee J.J. (2019). Performance of ^68^Ga-DOTA–Conjugated somatostatin receptor–targeting peptide PET in detection of pheochromocytoma and paraganglioma: A systematic review and metaanalysis. J. Nucl. Med..

[64] Taieb D., Hicks R.J., Hindie E., Guillet B.A., Avram A., Ghedini P., Timmers H.J., Scott A.T., Elojeimy S., Rubello D. (2019). European association of nuclear medicine practice guideline/society of nuclear medicine and molecular imaging procedure standard 2019 for radionuclide imaging of phaeochromocytoma and paraganglioma. Eur. J. Nucl. Med. Mol. Imaging.

[65] Jha A., de Luna K., Balili C.A., Millo C., Paraiso C.A., Ling A., Gonzales M.K., Viana B., Alrezk R., Adams K.T. (2019). Clinical, diagnostic, and treatment characteristics of SDHA-related metastatic pheochromocytoma and paraganglioma. Front. Oncol..

[66] Jha A., Ling A., Millo C., Chen C., Gupta G., Viana B., Gonzales M., Adams K., Herscovitch P., Lin F. (2018). Superiority of ^68^Ga-DOTATATE PET/CT to other functional and anatomic imaging modalities in the detection of SDHD-related pheochromocytoma and paraganglioma—A comparative prospective study. J. Nucl. Med..

[67] Jha A., Ling A., Millo C., Gupta G., Viana B., Lin F.I., Herscovitch P., Adams K.T., Taieb D., Metwalli A.R. (2018). Superiority of ^68^Ga-DOTATATE over ^18^F-FDG and anatomic imaging in the detection of succinate dehydrogenase mutation (SDHx)-related pheochromocytoma and paraganglioma in the pediatric population. Eur. J. Nucl. Med. Mol. Imaging.

[68] Janssen I., Chen C.C., Taieb D., Patronas N.J., Millo C.M., Adams K.T., Nambuba J., Herscovitch P., Sadowski S.M., Fojo A.T. (2016). ^68^Ga-DOTATATE PET/CT in the localization of head and neck paragangliomas compared with other functional imaging modalities and CT/MRI. J. Nucl. Med..

[69] Archier A., Varoquaux A., Garrigue P., Montava M., Guerin C., Gabriel S., Beschmout E., Morange I., Fakhry N., Castinetti F. (2016). Prospective comparison of ^68^Ga-DOTATATE and ^18^F-FDOPA PET/CT in patients with various pheochromocytomas and paragangliomas with emphasis on sporadic cases. Eur. J. Nucl. Med. Mol. Imaging.

[70] Kroiss A., Putzer D., Frech A., Decristoforo C., Uprimny C., Gasser R.W., Shulkin B.L., Url C., Widmann G., Prommegger R. (2013). A retrospective comparison between ^68^Ga-DOTA-TOC PET/CT and ^18^F-DOPA PET/CT in patients with extra-adrenal paraganglioma. Eur. J. Nucl. Med. Mol. Imaging.

[71] Janssen I., Chen C.C., Millo C.M., Ling A., Taieb D., Lin F.I., Adams K.T., Wolf K.I., Herscovitch P., Fojo A.T. (2016). PET/CT comparing ^68^Ga-DOTATATE and other radiopharmaceuticals and in comparison with CT/MRI for the localization of sporadic metastatic pheochromocytoma and paraganglioma. Eur. J. Nucl. Med. Mol. Imaging.

[72] Darr R., Nambuba J., Del Rivero J., Janssen I., Merino M., Todorovic M., Balint B., Jochmanova I., Prchal J.T., Lechan R.M. (2016). Novel insights into the polycythemia-paraganglioma-somatostatinoma syndrome. Endocr. Relat. Cancer.

[73] Janssen I., Chen C.C., Zhuang Z., Millo C.M., Wolf K.I., Ling A., Lin F.I., Adams K.T., Herscovitch P., Feelders R.A. (2017). Functional imaging signature of patients presenting with polycythemia/paraganglioma syndromes. J. Nucl. Med..

[74] Taieb D., Jha A., Guerin C., Pang Y., Adams K.T., Chen C.C., Romanet P., Roche P., Essamet W., Ling A. (2018). ^18^F-FDOPA PET/CT imaging of MAX-related pheochromocytoma. J. Clin. Endocrinol. Metab..

[75] Nambuba J., Därr R., Janssen I., Bullova P., Adams K.T., Millo C., Bourdeau I., Kassai A., Yang C., Kebebew E. (2015). Functional imaging experience in a germline fumarate hydratase mutation–positive patient with pheochromocytoma and paraganglioma. AACE Clin. Case Rep..

[76] Gild M.L., Naik N., Hoang J., Hsiao E., McGrath R.T., Sywak M., Sidhu S., Delbridge L.W., Robinson B.G., Schembri G. (2018). Role of DOTATATE-PET/CT in preoperative assessment of phaeochromocytoma and paragangliomas. Clin. Endocrinol..

[77] Fassnacht M., Arlt W., Bancos I., Dralle H., Newell-Price J., Sahdev A., Tabarin A., Terzolo M., Tsagarakis S., Dekkers O.M. (2016). Management of adrenal incidentalomas: European society of endocrinology clinical practice guideline in collaboration with the European network for the study of adrenal tumors. Eur. J. Endocrinol..

[78] Vanderveen K.A., Thompson S.M., Callstrom M.R., Young W.F., Grant C.S., Farley D.R., Richards M.L., Thompson G.B. (2009). Biopsy of pheochromocytomas and paragangliomas: Potential for disaster. Surgery.

[79] Dwight T., Flynn A., Amarasinghe K., Benn D.E., Lupat R., Li J., Cameron D.L., Hogg A., Balachander S., Candiloro I.L.M. (2018). TERT structural rearrangements in metastatic pheochromocytomas. Endocr. Relat. Cancer.

[80] Suh Y.J., Choe J.Y., Park H.J. (2017). Malignancy in pheochromocytoma or paraganglioma: Integrative analysis of 176 cases in TCGA. Endocr. Pathol..

[81] Stenman A., Svahn F., Hojjat-Farsangi M., Zedenius J., Soderkvist P., Gimm O., Larsson C., Juhlin C.C. (2019). Molecular profiling of pheochromocytoma and abdominal paraganglioma stratified by the PASS algorithm reveals chromogranin B as associated with histologic prediction of malignant behavior. Am. J. Surg. Pathol..

[82] Wang W., Zhong X., Ye L., Qi Y., Su T., Wei Q., Xie J., Jiang L., Jiang Y., Zhou W. (2016). ERBB-2 overexpression as a risk factor for malignant phaeochromocytomas and paraganglinomas. Clin. Endocrinol..

[83] Udager A.M., Magers M.J., Goerke D.M., Vinco M.L., Siddiqui J., Cao X., Lucas D.R., Myers J.L., Chinnaiyan A.M., McHugh J.B. (2018). The utility of SDHB and FH immunohistochemistry in patients evaluated for hereditary paraganglioma-pheochromocytoma syndromes. Hum. Pathol..

[84] O’Riordain D.S., Young W.F., Grant C.S., Carney J.A., van Heerden J.A. (1996). Clinical spectrum and outcome of functional extraadrenal paraganglioma. World J. Surg..

[85] King K.S., Prodanov T., Kantorovich V., Fojo T., Hewitt J.K., Zacharin M., Wesley R., Lodish M., Raygada M., Gimenez-Roqueplo A.P. (2011). Metastatic pheochromocytoma/paraganglioma related to primary tumor development in childhood or adolescence: Significant link to SDHB mutations. J. Clin. Oncol..

[86] Grossman A., Pacak K., Sawka A., Lenders J.W., Harlander D., Peaston R.T., Reznek R., Sisson J., Eisenhofer G. (2006). Biochemical diagnosis and localization of pheochromocytoma: Can we reach a consensus?. Ann. N. Y. Acad. Sci..

[87] Korevaar T.I., Grossman A.B. (2011). Pheochromocytomas and paragangliomas: Assessment of malignant potential. Endocrine.

[88] Bausch B., Wellner U., Bausch D., Schiavi F., Barontini M., Sanso G., Walz M.K., Peczkowska M., Weryha G., Dall’igna P. (2014). Long-term prognosis of patients with pediatric pheochromocytoma. Endocr. Relat. Cancer.

[89] Favier J., Amar L., Gimenez-Roqueplo A.P. (2015). Paraganglioma and phaeochromocytoma: From genetics to personalized medicine. Nat. Rev. Endocrinol..

[90] Wells S.A., Asa S.L., Dralle H., Elisei R., Evans D.B., Gagel R.F., Lee N., Machens A., Moley J.F., Pacini F. (2015). Revised American Thyroid Association guidelines for the management of medullary thyroid carcinoma. Thyroid.

[91] Nielsen S.M., Rhodes L., Blanco I., Chung W.K., Eng C., Maher E.R., Richard S., Giles R.H. (2016). Von Hippel-Lindau disease: Genetics and role of genetic counseling in a multiple neoplasia syndrome. J. Clin. Oncol..

[92] Rednam S.P., Erez A., Druker H., Janeway K.A., Kamihara J., Kohlmann W.K., Nathanson K.L., States L.J., Tomlinson G.E., Villani A. (2017). Von Hippel-Lindau and hereditary pheochromocytoma/paraganglioma syndromes: Clinical features, genetics, and surveillance recommendations in childhood. Clin. Cancer Res..

[93] Gutmann D.H., Aylsworth A., Carey J.C., Korf B., Marks J., Pyeritz R.E., Rubenstein A., Viskochil D. (1997). The diagnostic evaluation and multidisciplinary management of neurofibromatosis 1 and neurofibromatosis 2. JAMA.

[94] Brandi M.L., Gagel R.F., Angeli A., Bilezikian J.P., Beck-Peccoz P., Bordi C., Conte-Devolx B., Falchetti A., Gheri R.G., Libroia A. (2001). Guidelines for diagnosis and therapy of MEN type 1 and type 2. J. Clin. Endocrinol. Metab..

[95] Buffet A., Ben Aim L., Leboulleux S., Drui D., Vezzosi D., Libe R., Ajzenberg C., Bernardeschi D., Cariou B., Chabolle F. (2019). Positive impact of genetic test on the management and outcome of patients with paraganglioma and/or pheochromocytoma. J. Clin. Endocrinol. Metab..

[96] Walz M.K., Alesina P.F., Wenger F.A., Deligiannis A., Szuczik E., Petersenn S., Ommer A., Groeben H., Peitgen K., Janssen O.E. (2006). Posterior retroperitoneoscopic adrenalectomy—Results of 560 procedures in 520 patients. Surgery.

[97] Castinetti F., Qi X.P., Walz M.K., Maia A.L., Sanso G., Peczkowska M., Hasse-Lazar K., Links T.P., Dvorakova S., Toledo R.A. (2014). Outcomes of adrenal-sparing surgery or total adrenalectomy in phaeochromocytoma associated with multiple endocrine neoplasia type 2: An international retrospective population-based study. Lancet Oncol..

[98] Moore M.G., Netterville J.L., Mendenhall W.M., Isaacson B., Nussenbaum B. (2016). Head and neck paragangliomas: An update on evaluation and management. Otolaryngol. Head Neck Surg..

[99] Capatina C., Ntali G., Karavitaki N., Grossman A.B. (2013). The management of head-and-neck paragangliomas. Endocr. Relat. Cancer.

[100] Marchetti M., Pinzi V., Tramacere I., Bianchi L.C., Ghielmetti F., Fariselli L. (2017). Radiosurgery for paragangliomas of the head and neck: Another step for the validation of a treatment paradigm. World Neurosurg..

[101] Dupin C., Lang P., Dessard-Diana B., Simon J.M., Cuenca X., Mazeron J.J., Feuvret L. (2014). Treatment of head and neck paragangliomas with external beam radiation therapy. Int. J. Radiat. Oncol. Biol. Phys..

[102] Vogel J., Atanacio A.S., Prodanov T., Turkbey B.I., Adams K., Martucci V., Camphausen K., Fojo A.T., Pacak K., Kaushal A. (2014). External beam radiation therapy in treatment of malignant pheochromocytoma and paraganglioma. Front. Oncol..

[103] Neumann H.P.H., Tsoy U., Bancos I., Amodru V., Walz M.K., Tirosh A., Kaur R.J., McKenzie T., Qi X., Bandgar T. (2019). Comparison of pheochromocytoma-specific morbidity and mortality among adults with bilateral pheochromocytomas undergoing total adrenalectomy vs cortical-sparing adrenalectomy. JAMA Netw. Open.

[104] Roman-Gonzalez A., Zhou S., Ayala-Ramirez M., Shen C., Waguespack S.G., Habra M.A., Karam J.A., Perrier N., Wood C.G., Jimenez C. (2017). Impact of surgical resection of the primary tumor on overall survival in patients with metastatic pheochromocytoma or sympathetic paraganglioma. Ann. Surg..

[105] Ayala-Ramirez M., Feng L., Habra M.A., Rich T., Dickson P.V., Perrier N., Phan A., Waguespack S., Patel S., Jimenez C. (2012). Clinical benefits of systemic chemotherapy for patients with metastatic pheochromocytomas or sympathetic extra-adrenal paragangliomas: Insights from the largest single-institutional experience. Cancer.

[106] Hamidi O., Young W.F., Iniguez-Ariza N.M., Kittah N.E., Gruber L., Bancos C., Tamhane S., Bancos I. (2017). Malignant pheochromocytoma and paraganglioma: 272 patients over 55 years. J. Clin. Endocrinol. Metab..

[107] Strajina V., Dy B.M., Farley D.R., Richards M.L., McKenzie T.J., Bible K.C., Que F.G., Nagorney D.M., Young W.F., Thompson G.B. (2017). Surgical treatment of malignant pheochromocytoma and paraganglioma: Retrospective case series. Ann. Surg. Oncol..

[108] Wei S., Wu D., Yue J. (2013). Surgical resection of multiple liver metastasis of functional malignant pheochromocytoma: A case report and literature review. J. Cancer Res. Ther..

[109] Arnas-Leon C., Sanchez V., Santana Suarez A.D., Quintana Arroyo S., Acosta C., Martinez Martin F.J. (2016). Complete remission in metastatic pheochromocytoma treated with extensive surgery. Cureus.

[110] Breen W., Bancos I., Young W.F., Bible K.C., Laack N.N., Foote R.L., Hallemeier C.L. (2018). External beam radiation therapy for advanced/unresectable malignant paraganglioma and pheochromocytoma. Adv. Radiat. Oncol..

[111] Gravel G., Leboulleux S., Tselikas L., Fassio F., Berraf M., Berdelou A., Ba B., Hescot S., Hadoux J., Schlumberger M. (2018). Prevention of serious skeletal-related events by interventional radiology techniques in patients with malignant paraganglioma and pheochromocytoma. Endocrine.

[112] Kohlenberg J., Welch B., Hamidi O., Callstrom M., Morris J., Sprung J., Bancos I., Young W. (2019). Efficacy and safety of ablative therapy in the treatment of patients with metastatic pheochromocytoma and paraganglioma. Cancers.

[113] Plouin P.F., Duclos J.M., Soppelsa F., Boublil G., Chatellier G. (2001). Factors associated with perioperative morbidity and mortality in patients with pheochromocytoma: Analysis of 165 operations at a single center. J. Clin. Endocrinol. Metab..

[114] Pacak K. (2007). Preoperative management of the pheochromocytoma patient. J. Clin. Endocrinol. Metab..

[115] van der Zee P.A., de Boer A. (2014). Pheochromocytoma: A review on preoperative treatment with phenoxybenzamine or doxazosin. Neth. J. Med..

[116] Mak I.Y.F., Hayes A.R., Khoo B., Grossman A. (2019). Peptide receptor radionuclide therapy as a novel treatment for metastatic and invasive phaeochromocytoma and paraganglioma. Neuroendocrinology.

[117] Pryma D.A., Chin B.B., Noto R.B., Dillon J.S., Perkins S., Solnes L., Kostakoglu L., Serafini A.N., Pampaloni M.H., Jensen J. (2019). Efficacy and safety of high-specific-activity ^131^I-MIBG therapy in patients with advanced pheochromocytoma or paraganglioma. J. Nucl. Med..

[118] van Hulsteijn L.T., Niemeijer N.D., Dekkers O.M., Corssmit E.P. (2014). ^131^I-MIBG therapy for malignant paraganglioma and phaeochromocytoma: Systematic review and meta-analysis. Clin. Endocrinol..

[119] Castellani M.R., Seghezzi S., Chiesa C., Aliberti G.L., Maccauro M., Seregni E., Orunesu E., Luksch R., Bombardieri E. (2010). ^131^I-MIBG treatment of pheochromocytoma: Low versus intermediate activity regimens of therapy. Q. J. Nucl. Med. Mol. Imaging.

[120] Sze W.C., Grossman A.B., Goddard I., Amendra D., Shieh S.C., Plowman P.N., Drake W.M., Akker S.A., Druce M.R. (2013). Sequelae and survivorship in patients treated with ^131^I-MIBG therapy. Br. J. Cancer.

[121] Ziegler C.G., Brown J.W., Schally A.V., Erler A., Gebauer L., Treszl A., Young L., Fishman L.M., Engel J.B., Willenberg H.S. (2009). Expression of neuropeptide hormone receptors in human adrenal tumors and cell lines: Antiproliferative effects of peptide analogues. Proc. Natl. Acad. Sci. USA.

[122] van Essen M., Krenning E.P., de Jong M., Valkema R., Kwekkeboom D.J. (2007). Peptide receptor radionuclide therapy with radiolabelled somatostatin analogues in patients with somatostatin receptor positive tumours. Acta Oncol..

[123] van Essen M., Krenning E.P., Kooij P.P., Bakker W.H., Feelders R.A., de Herder W.W., Wolbers J.G., Kwekkeboom D.J. (2006). Effects of therapy with [^177^Lu-DOTA^0^, Tyr^3^] octreotate in patients with paraganglioma, meningioma, small cell lung carcinoma, and melanoma. J. Nucl. Med..

[124] Zovato S., Kumanova A., Dematte S., Sansovini M., Bodei L., Di Sarra D., Casagranda E., Severi S., Ambrosetti A., Schiavi F. (2012). Peptide receptor radionuclide therapy (PRRT) with ^177^Lu-DOTATATE in individuals with neck or mediastinal paraganglioma (PGL). Horm. Metab. Res..

[125] Forrer F., Riedweg I., Maecke H.R., Mueller-Brand J. (2008). Radiolabeled DOTATOC in patients with advanced paraganglioma and pheochromocytoma. Q. J. Nucl. Med. Mol. Imaging.

[126] Kong G., Grozinsky-Glasberg S., Hofman M.S., Callahan J., Meirovitz A., Maimon O., Pattison D.A., Gross D.J., Hicks R.J. (2017). Efficacy of peptide receptor radionuclide therapy for functional metastatic paraganglioma and pheochromocytoma. J. Clin. Endocrinol. Metab..

[127] Nastos K., Cheung V.T.F., Toumpanakis C., Navalkissoor S., Quigley A.M., Caplin M., Khoo B. (2017). Peptide receptor radionuclide treatment and ^131^I-MIBG in the management of patients with metastatic/progressive phaeochromocytomas and paragangliomas. J. Surg. Oncol..

[128] Pinato D.J., Black J.R., Ramaswami R., Tan T.M., Adjogatse D., Sharma R. (2016). Peptide receptor radionuclide therapy for metastatic paragangliomas. Med. Oncol..

[129] Imhof A., Brunner P., Marincek N., Briel M., Schindler C., Rasch H., Macke H.R., Rochlitz C., Muller-Brand J., Walter M.A. (2011). Response, survival, and long-term toxicity after therapy with the radiolabeled somatostatin analogue [^90^Y-DOTA]-TOC in metastasized neuroendocrine cancers. J. Clin. Oncol..

[130] Puranik A.D., Kulkarni H.R., Singh A., Baum R.P. (2015). Peptide receptor radionuclide therapy with ^90^Y/^177^Lu-labelled peptides for inoperable head and neck paragangliomas (glomus tumours). Eur. J. Nucl. Med. Mol. Imaging.

[131] Yadav M.P., Ballal S., Bal C. (2019). Concomitant ^177^Lu-DOTATATE and capecitabine therapy in malignant paragangliomas. EJNMMI Res..

[132] Estevao R., Duarte H., Lopes F., Fernandes J., Monteiro E. (2015). Peptide receptor radionuclide therapy in head and neck paragangliomas—Report of 14 cases. Rev. Laryngol. Otol. Rhinol..

[133] Strosberg J., El-Haddad G., Wolin E., Hendifar A., Yao J., Chasen B., Mittra E., Kunz P.L., Kulke M.H., Jacene H. (2017). Phase 3 trial of ^177^Lu-DOTATATE for midgut neuroendocrine tumors. N. Engl. J. Med..

[134] Navalkissoor S., Grossman A. (2019). Targeted alpha particle therapy for neuroendocrine tumours: The next generation of peptide receptor radionuclide therapy. Neuroendocrinology.

[135] Brabander T., van der Zwan W.A., Teunissen J.J.M., Kam B.L.R., Feelders R.A., de Herder W.W., van Eijck C.H.J., Franssen G.J.H., Krenning E.P., Kwekkeboom D.J. (2017). Long-term efficacy, survival, and safety of [^177^Lu-DOTA^0^,Tyr^3^]octreotate in patients with gastroenteropancreatic and bronchial neuroendocrine tumors. Clin. Cancer Res..

[136] Bodei L., Cremonesi M., Kidd M., Grana C.M., Severi S., Modlin I.M., Paganelli G. (2014). Peptide receptor radionuclide therapy for advanced neuroendocrine tumors. Thorac. Surg. Clin..

[137] Bodei L., Cremonesi M., Paganelli G. (2014). Yttrium-based therapy for neuroendocrine tumors. PET Clin..

[138] Bergsma H., van Lom K., Raaijmakers M., Konijnenberg M., Kam B., Teunissen J.J.M., de Herder W.W., Krenning E.P., Kwekkeboom D.J. (2018). Persistent hematologic dysfunction after peptide receptor radionuclide therapy with ^177^Lu-DOTATATE: Incidence, course, and predicting factors in patients with gastroenteropancreatic neuroendocrine tumors. J. Nucl. Med..

[139] Schuchardt C., Kulkarni H.R., Prasad V., Zachert C., Muller D., Baum R.P. (2013). The Bad Berka dose protocol: Comparative results of dosimetry in peptide receptor radionuclide therapy using ^177^Lu-DOTATATE, ^177^Lu-DOTANOC, and ^177^Lu-DOTATOC. Theranostics, Gallium-68, and Other Radionuclides.

[140] Del Prete M., Buteau F.A., Arsenault F., Saighi N., Bouchard L.O., Beaulieu A., Beauregard J.M. (2019). Personalized ^177^Lu-octreotate peptide receptor radionuclide therapy of neuroendocrine tumours: Initial results from the P-PRRT trial. Eur. J. Nucl. Med. Mol. Imaging.

[141] Taieb D., Garrigue P., Bardies M., Abdullah A.E., Pacak K. (2015). Application and dosimetric requirements for gallium-68–labeled somatostatin analogues in targeted radionuclide therapy for gastroenteropancreatic neuroendocrine tumors. PET Clin..

[142] Huizing D.M.V., de Wit-van der Veen B.J., Verheij M., Stokkel M.P.M. (2018). Dosimetry methods and clinical applications in peptide receptor radionuclide therapy for neuroendocrine tumours: A literature review. EJNMMI Res..

[143] Rinke A., Muller H.H., Schade-Brittinger C., Klose K.J., Barth P., Wied M., Mayer C., Aminossadati B., Pape U.F., Blaker M. (2009). Placebo-controlled, double-blind, prospective, randomized study on the effect of octreotide LAR in the control of tumor growth in patients with metastatic neuroendocrine midgut tumors: A report from the PROMID Study Group. J. Clin. Oncol..

[144] Caplin M.E., Pavel M., Cwikla J.B., Phan A.T., Raderer M., Sedlackova E., Cadiot G., Wolin E.M., Capdevila J., Wall L. (2014). Lanreotide in metastatic enteropancreatic neuroendocrine tumors. N. Engl. J. Med..

[145] Niemeijer N.D., Alblas G., van Hulsteijn L.T., Dekkers O.M., Corssmit E.P. (2014). Chemotherapy with cyclophosphamide, vincristine and dacarbazine for malignant paraganglioma and pheochromocytoma: Systematic review and meta-analysis. Clin. Endocrinol..

[146] Averbuch S.D., Steakley C.S., Young R.C., Gelmann E.P., Goldstein D.S., Stull R., Keiser H.R. (1988). Malignant pheochromocytoma: Effective treatment with a combination of cyclophosphamide, vincristine, and dacarbazine. Ann. Inter. Med..

[147] Huang H., Abraham J., Hung E., Averbuch S., Merino M., Steinberg S.M., Pacak K., Fojo T. (2008). Treatment of malignant pheochromocytoma/paraganglioma with cyclophosphamide, vincristine, and dacarbazine: Recommendation from a 22-year follow-up of 18 patients. Cancer.

[148] Jawed I., Velarde M., Darr R., Wolf K.I., Adams K., Venkatesan A.M., Balasubramaniam S., Poruchynsky M.S., Reynolds J.C., Pacak K. (2018). Continued tumor reduction of metastatic pheochromocytoma/paraganglioma harboring succinate dehydrogenase subunit B mutations with cyclical chemotherapy. Cell. Mol. Neurobiol..

[149] Hadoux J., Favier J., Scoazec J.Y., Leboulleux S., Al Ghuzlan A., Caramella C., Deandreis D., Borget I., Loriot C., Chougnet C. (2014). SDHB mutations are associated with response to temozolomide in patients with metastatic pheochromocytoma or paraganglioma. Int. J. Cancer.

[150] Tena I., Gupta G., Tajahuerce M., Benavent M., Cifrian M., Falcon A., Fonfria M., Del Olmo M., Reboll R., Conde A. (2018). Successful second-line metronomic temozolomide in metastatic paraganglioma: Case reports and review of the literature. Clin. Med. Insights Oncol..

[151] Pegg A.E., Dolan M.E., Moschel R.C. (1995). Structure, function, and inhibition of O^6^-alkylguanine-DNA alkyltransferase. Progress in Nucleic Acid Research and Molecular Biology.

[152] Hegi M.E., Liu L., Herman J.G., Stupp R., Wick W., Weller M., Mehta M.P., Gilbert M.R. (2008). Correlation of O^6^-methylguanine methyltransferase (MGMT) promoter methylation with clinical outcomes in glioblastoma and clinical strategies to modulate MGMT activity. J. Clin. Oncol..

[153] Bignami M., O’Driscoll M., Aquilina G., Karran P. (2000). Unmasking a killer: DNA O^6^-methylguanine and the cytotoxicity of methylating agents. Mutat. Res..

[154] Tay C.G., Lee V.W.M., Ong L.C., Goh K.J., Ariffin H., Fong C.Y. (2017). Vincristine-induced peripheral neuropathy in survivors of childhood acute lymphoblastic leukaemia. Pediatric Blood Cancer.

[155] Ayala-Ramirez M., Chougnet C.N., Habra M.A., Palmer J.L., Leboulleux S., Cabanillas M.E., Caramella C., Anderson P., Al Ghuzlan A., Waguespack S.G. (2012). Treatment with sunitinib for patients with progressive metastatic pheochromocytomas and sympathetic paragangliomas. J. Clin. Endocrinol. Metab..

[156] O’Kane G.M., Ezzat S., Joshua A.M., Bourdeau I., Leibowitz-Amit R., Olney H.J., Krzyzanowska M., Reuther D., Chin S., Wang L. (2019). A phase 2 trial of sunitinib in patients with progressive paraganglioma or pheochromocytoma: The SNIPP trial. Br. J. Cancer.

[157] Choueiri T.K., Halabi S., Sanford B.L., Hahn O., Michaelson M.D., Walsh M.K., Feldman D.R., Olencki T., Picus J., Small E.J. (2017). Cabozantinib versus sunitinib as initial targeted therapy for patients with metastatic renal cell carcinoma of poor or intermediate risk: The Alliance A031203 CABOSUN trial. J. Clin. Oncol..

[158] Choueiri T.K., Hessel C., Halabi S., Sanford B., Michaelson M.D., Hahn O., Walsh M., Olencki T., Picus J., Small E.J. (2018). Cabozantinib versus sunitinib as initial therapy for metastatic renal cell carcinoma of intermediate or poor risk (Alliance A031203 CABOSUN randomised trial): Progression-free survival by independent review and overall survival update. Eur. J. Cancer.

[159] Fankhauser M., Bechmann N., Lauseker M., Goncalves J., Favier J., Klink B., William D., Gieldon L., Maurer J., Spottl G. (2019). Synergistic highly potent targeted drug combinations in different pheochromocytoma models including human tumor cultures. Endocrinology.

[160] Jimenez P., Tatsui C., Jessop A., Thosani S., Jimenez C. (2017). Treatment for malignant pheochromocytomas and paragangliomas: 5 years of progress. Curr. Oncol. Rep..

[161] Jasim S., Suman V.J., Jimenez C., Harris P., Sideras K., Burton J.K., Worden F.P., Auchus R.J., Bible K.C. (2017). Phase II trial of pazopanib in advanced/progressive malignant pheochromocytoma and paraganglioma. Endocrine.

[162] Oh D.Y., Kim T.W., Park Y.S., Shin S.J., Shin S.H., Song E.K., Lee H.J., Lee K.W., Bang Y.J. (2012). Phase 2 study of everolimus monotherapy in patients with nonfunctioning neuroendocrine tumors or pheochromocytomas/paragangliomas. Cancer.

[163] Druce M.R., Kaltsas G.A., Fraenkel M., Gross D.J., Grossman A.B. (2009). Novel and evolving therapies in the treatment of malignant phaeochromocytoma: Experience with the mTOR inhibitor everolimus (RAD001). Horm. Metab. Res..

[164] Nölting S., Garcia E., Alusi G., Giubellino A., Pacak K., Korbonits M., Grossman A.B. (2012). Combined blockade of signalling pathways shows marked anti-tumour potential in phaeochromocytoma cell lines. J. Mol. Endocrinol..

[165] Nölting S., Giubellino A., Tayem Y., Young K., Lauseker M., Bullova P., Schovanek J., Anver M., Fliedner S., Korbonits M. (2014). Combination of 13-Cis retinoic acid and lovastatin: Marked anti-tumor potential in vivo in a pheochromocytoma allograft model in female athymic nude mice. Endocrinology.

[166] Nölting S., Maurer J., Spottl G., Aristizabal Prada E.T., Reuther C., Young K., Korbonits M., Goke B., Grossman A., Auernhammer C.J. (2015). Additive anti-tumor effects of lovastatin and everolimus in vitro through simultaneous inhibition of signaling pathways. PLoS ONE.

[167] Giubellino A., Bullova P., Nolting S., Turkova H., Powers J.F., Liu Q., Guichard S., Tischler A.S., Grossman A.B., Pacak K. (2013). Combined inhibition of mTORC1 and mTORC2 signaling pathways is a promising therapeutic option in inhibiting pheochromocytoma tumor growth: In vitro and in vivo studies in female athymic nude mice. Endocrinology.

[168] Pang Y., Liu Y., Pacak K., Yang C. (2019). Pheochromocytomas and paragangliomas: From genetic diversity to targeted therapies. Cancers.

[169] Hatfield S.M., Sitkovsky M. (2016). A2A adenosine receptor antagonists to weaken the hypoxia-HIF-1alpha driven immunosuppression and improve immunotherapies of cancer. Curr. Opin. Pharmacol..

[170] Labiano S., Palazon A., Bolanos E., Azpilikueta A., Sanchez-Paulete A.R., Morales-Kastresana A., Quetglas J.I., Perez-Gracia J.L., Gurpide A., Rodriguez-Ruiz M. (2016). Hypoxia-induced soluble CD137 in malignant cells blocks CD137L-costimulation as an immune escape mechanism. Oncoimmunology.

[171] Chouaib S., Noman M.Z., Kosmatopoulos K., Curran M.A. (2017). Hypoxic stress: Obstacles and opportunities for innovative immunotherapy of cancer. Oncogene.

